# Supplementary material for: Transcriptomic analysis of intestine following administration of a transglutaminase 2 inhibitor to prevent gluten-induced intestinal damage in celiac disease
Source: Nat Immunol. 2024 Jun 24;25(7):1218–30. doi: 10.1038/s41590-024-01867-0 (PMC11224021; doi:10.1038/s41590-024-01867-0)
Supplement: Supplementary file 1 — Supplementary Tables 1 and 2 and Figs. 1–5. [file 41590_2024_1867_MOESM1_ESM.pdf]

nature immunology

Supplementary information https://doi.org/10.1038/s41590-024-01867-0

Transcriptomic analysis of intestine following administration of a transglutaminase 2 inhibitor to prevent gluten-induced intestinal damage in celiac disease

In the format provided by the authors and unedited

Supplementary Files

Supplementary Table 1: Demographic Characteristics of the Patients in original cohort and in present study.

Supplementary Table 2: Effect of ZED1227 Treatment on the Ratio of villus height to crypt depth (VH:CrD) in original cohort and in present study.

Supplementary Fig. 1: Expression of common genes.

Supplementary Fig. 2: Transcriptional factors enrichment.

Supplementary Fig. 3: Cell type proportions according to the results of duodenal biopsies bulk transcriptomics deconvolution.

Supplementary Fig. 4: Immune Signaling Pathways, TG2 mRNA levels and TG2 activity during ZED1227 treatment.

Supplementary Fig. 5: ZED1227 treatment of human intestinal organoids.

Supplementary Table 1: Demographic Characteristics of the Patients in original cohort and in present study.

	Original cohort*		Present study cohort	
Characteristic	ZED1227, 100 mg (N = 39)	Placebo (N = 38)	Drug (d, n=34)	Placebo (p, n=24)
Age — yr (mean ± sd)	41.0±14.8	42.5±14.4	40.7±15.1	43.2±14.9
Female sex — no. (%)	24 (62)	28 (74)	22 (64.7)	17 (70.8)
White race — no. (%)	39 (100)	38 (100)	34 (100)	24 (100)
Weight — kg (mean ± sd)	73.2±13.7	68.4±14.7	74.1±13.9	71.4±17.0

*Original cohort Demographic Characteristics is published in Schuppan, D. et al. A Randomized Trial of a Transglutaminase 2 Inhibitor for Celiac Disease. N. Engl. J. Med. 385, 35–45 (2021).

Supplementary Table 2: Effect of ZED1227 Treatment on the Ratio of villus height to crypt depth (VH:CrD) in original cohort and in present study.

	Original cohort*		Present study cohort	
Variable	ZED1227, 100 mg (N = 38)	Placebo (N = 30)	Drug (d, n=34)	Placebo (p, n=24)
VH:CrD				
GFD	2.09±0.35	1.98±0.33	2.11±0.34	1.95±0.36
PGC	1.94±0.48	1.39±0.61	1.89±0.40	1.35±0.65
Change from GFD (95% CI)	−0.13 (−0.28 to 0.03)	−0.61 (−0.78 to −0.44)	−0.21 (−0.36 to -0.07)	−0.59 (−0.83 to −0.35)

*Original cohort VH:CrD descriptive statistics is published in Schuppan, D. et al. A Randomized Trial of a Transglutaminase 2 Inhibitor for Celiac Disease. N. Engl. J. Med. 385, 35–45 (2021).

Plus–minus values are means ±SD. The change from GFD is presented as a least-squares means estimate.

Supplementary Fig. 1

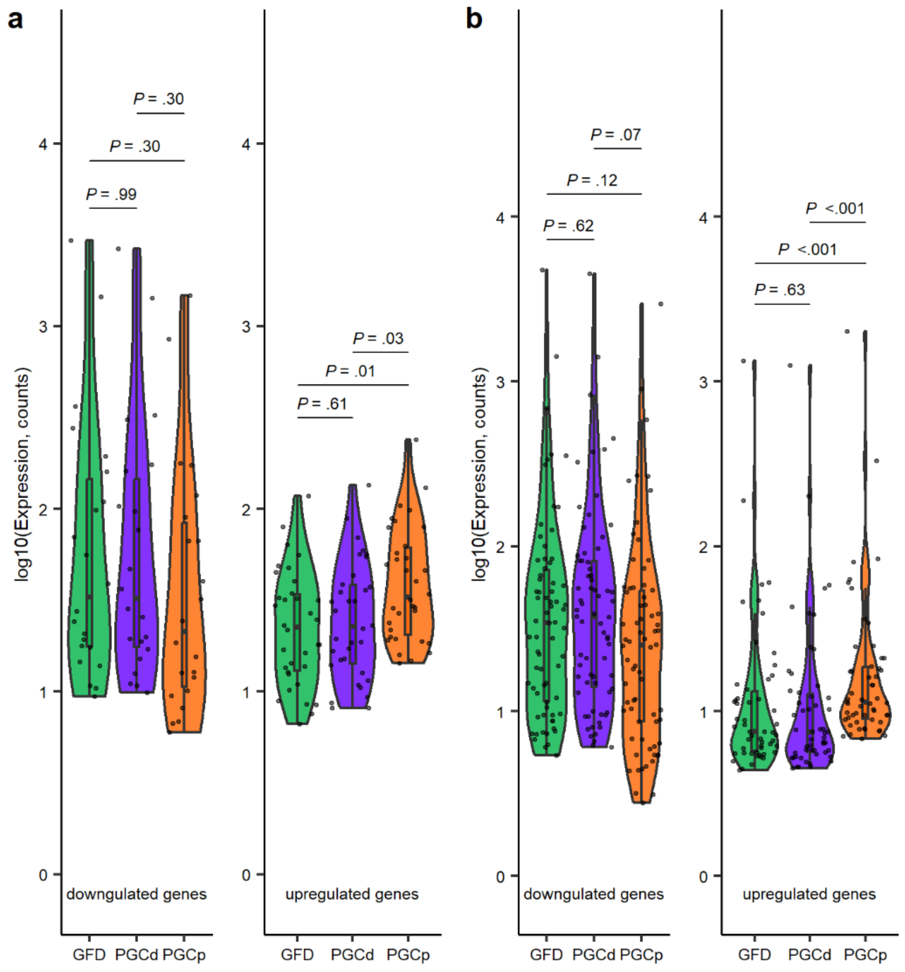

Supplementary Fig. 1: Expression of common genes. a, Violin plots showing the log-transformed expression of 56 common (in PGCp VS PGCd and PGCp VS GFDp comparisons) DEGs down-regulated (left panel) and up-regulated (right panel). **b,** Violin plot of 124 uniquely differentially expressed genes in PGCp VS PGCd comparison'. Kruskal-Wallis test followed by the Dunn's post hoc test was used for group comparisons. Statistical significance was defined as a $P < .05$. **a**: downregulated genes: GFD-PGCd $P = 0.99$, PGCp-GFD $P = 0.30$, PGCd-PGCp P= 0.30; upregulated genes: GFD-PGCd $P = 0.61$, PGCp-GFD $P = 0.01$, PGCd-PGCp P= 0.03; **b**: downregulated genes: GFD-PGCd $P = 0.62$, PGCp-GFD $P = 0.12$, PGCd-PGCp P= 0.07; upregulated genes: GFD-PGCd $P = 0.63$, PGCp-GFD $P = 6.68 \times 10^{-4}$, PGCd-PGCp P= 1.98×10^{-4}. The box plot center lines represent the median, the box boundaries represent IQR and the whisker length minimum and maximum range. Values from individual patients are shown. GFD (n = 58), PGCd (n = 34), and PGCp (n = 23)

Supplementary Fig. 2

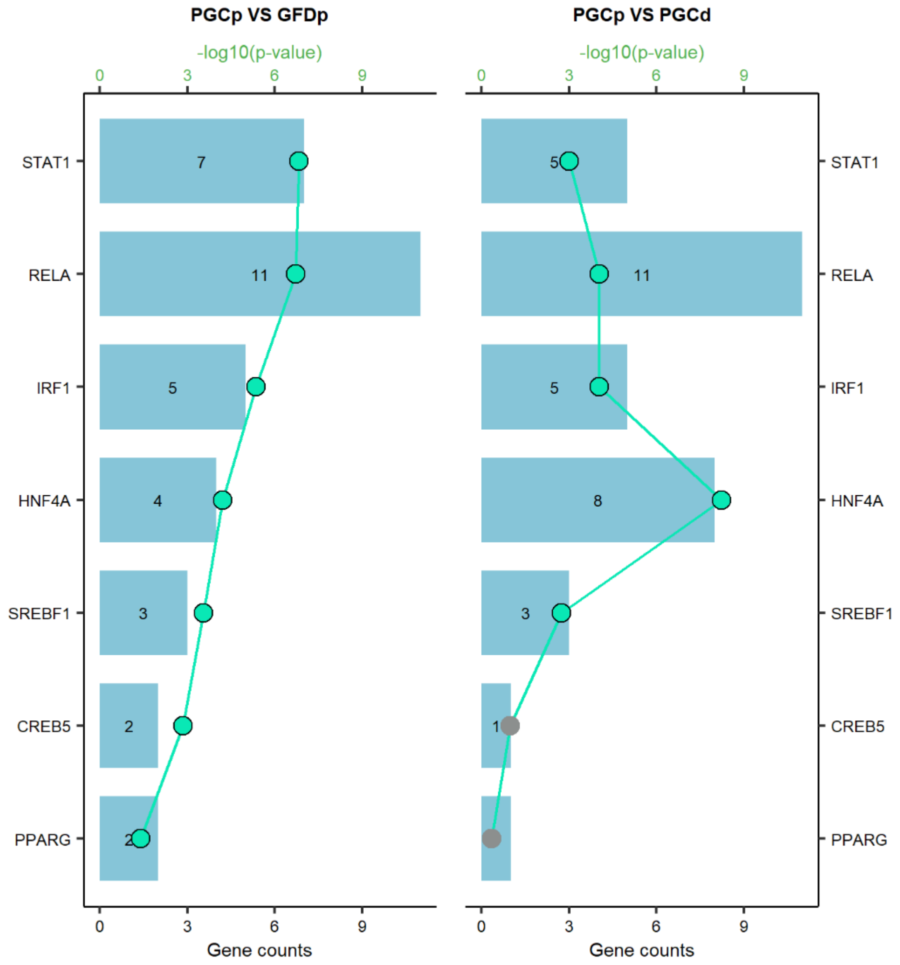

Supplementary Fig. 2: Transcriptional factors enrichment. Barplot of enriched transcriptional factors in PGCp vs GFDp (left) and PGCp vs. PGCd (right) comparisons. Fisher's exact test was used for p-values calculation. Green and gray dots denote significant and non-significant adjusted p-values, respectively. Statistical significance was defined as a $P < .05$ ($-\log10(\text{p-value}) > 1.3$).

Supplementary Fig. 3

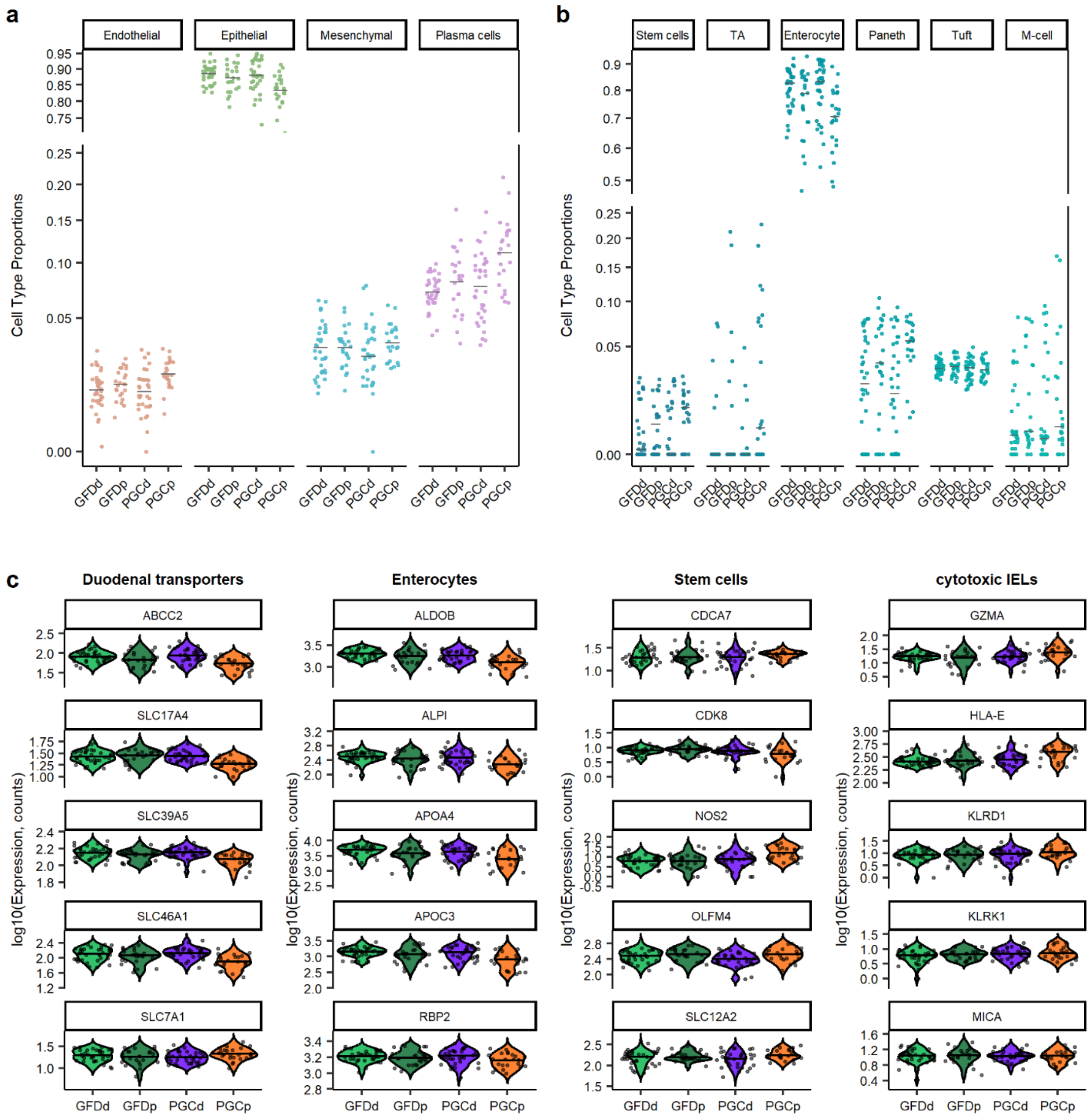

Supplementary Fig. 3: Cell type proportions according to the results of duodenal biopsies bulk transcriptomics deconvolution for cell category **a,** and cell type **b,** for patients in drug and placebo groups on GFD and at PGC (GFDd, n = 34; GFDp, n = 24; PGCd, n = 34; PGCp, n = 23). The horizontal line represents mean. **c,** Violin plots for selected genes expression. Midline denotes the median. GFDd, n = 34; GFDp, n = 24; PGCd, n = 34; PGCp, n = 23.

Supplementary Fig. 4

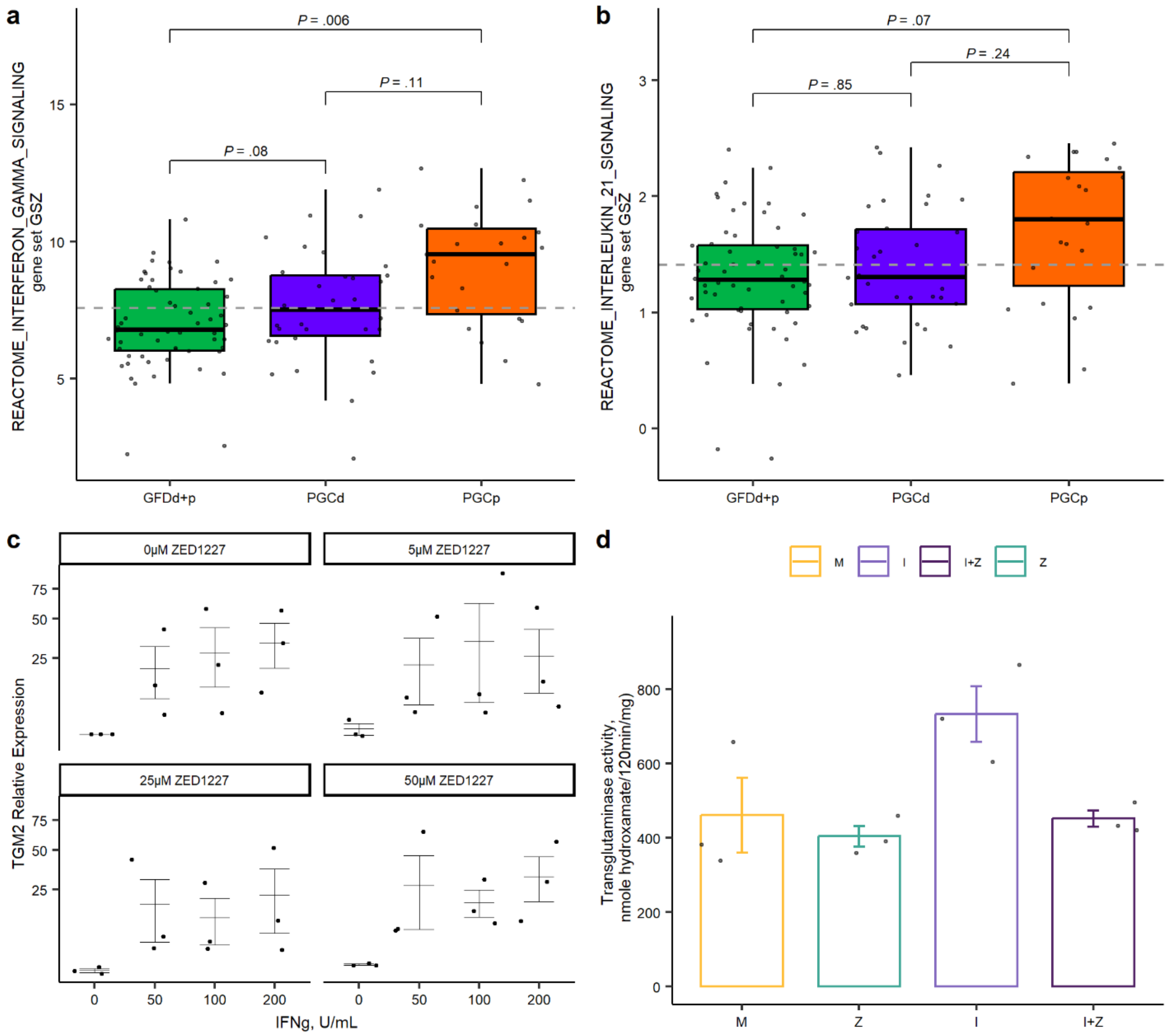

Supplementary Fig. 4: Immune Signaling Pathways, TG2 mRNA levels and TG2 activity during ZED1227 treatment. a, Gene set Z-score analyses for the Reactome pathway database gene set "Interferon-gamma signaling" gene set. **b,** Gene set Z-score analyses for the Reactome pathway database gene set "Interleukin-21 signaling" gene set. In **a** and **b,** GSZ scores were compared among groups using asymptotic p-value estimation, with statistical significance defined as $P < .05$. The box plot center lines represent the median, the box boundaries represent IQR and the whisker length minimum and maximum range. Values from individual patients are shown. GFDd+p (n = 58), PGCd (n = 34), and PGCp (n = 23). **c,** Quantitative RT-PCR of TGM2 expression in human duodenal organoids (n = 3) treated with human recombinant IFNg and/or ZED1227 at specified concentrations. Gene-specific Ct values were normalized (ΔΔCt) based on GAPDH house-keeping gene expression and relative to non-treated sample (0 U/mL IFNg and 0 mM ZED1227). Data shown as mean ± SE. **d,** Colorimetric TG activity assay in Caco2 cells treated with 100 U/mL IFNɣ (I, n = 3), 50 µM ZED1227 (Z, n = 3), their combination (I+Z, n = 3), or mock (M, n = 3) for 24 h. Data represented as mean ± SE.

Supplementary Fig. 5

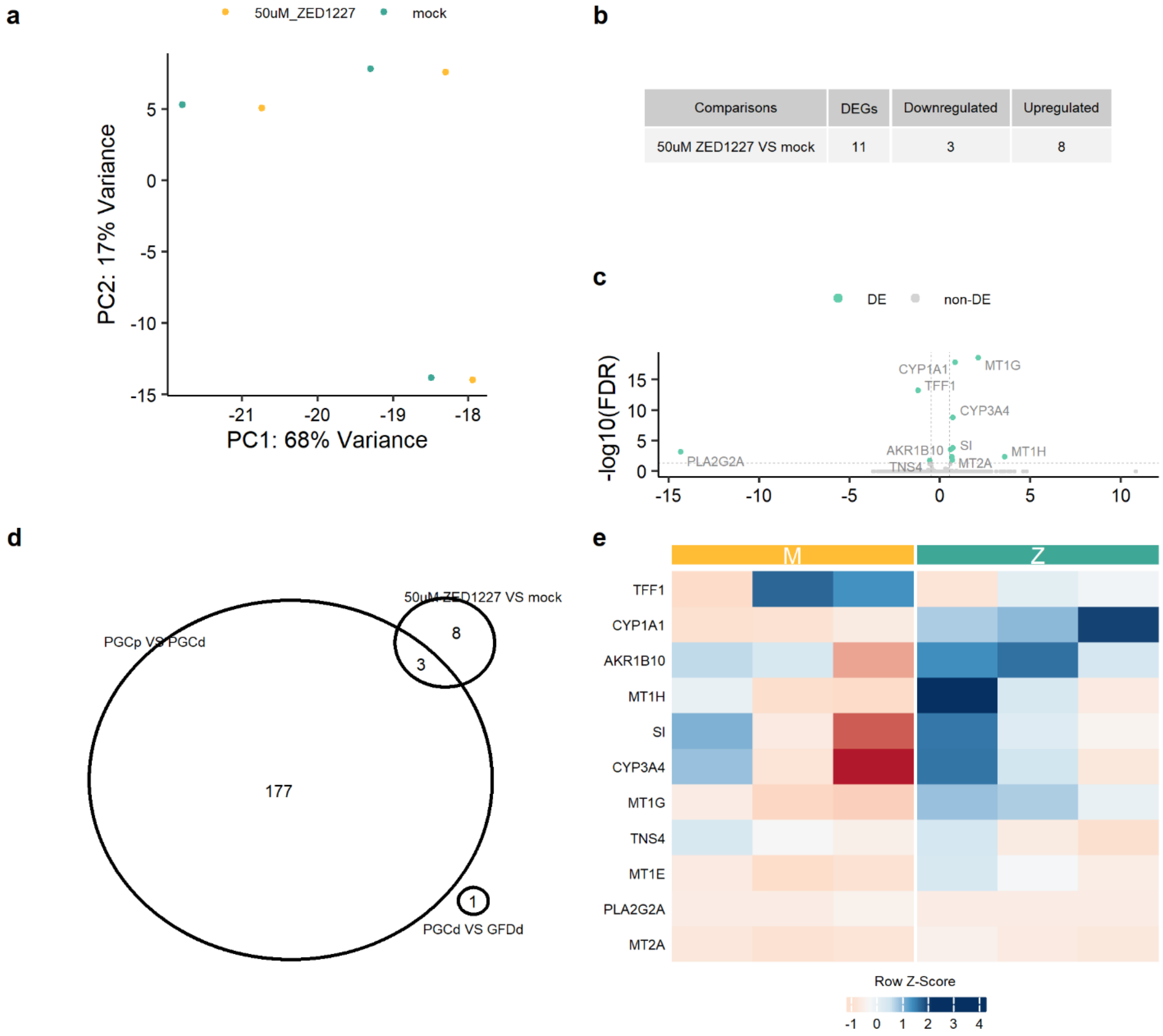

Supplementary Fig. 5: ZED1227 treatment of human intestinal organoids. a, Principal component analysis (PCA) plot using DESeq2-transformed counts for all organoid samples (n = 3), treated with 50µM ZED1227 (n = 3) or mock-treated (n = 3) for 24 hours. **b,** Table showing the number of differentially expressed genes (DEGs) (log2FC ≥ |0.5| and FDR ≤ .05) in the 50µM ZED1227 VS mock comparison. **c,** Volcano plot representations of DEGs (n = 11) in 50µM ZED1227 VS mock comparison. The green dots indicate DEGs (FDR ≤ 0.05) above the threshold (log2FC ≥ 0.5 and ≤ -0.5). The dashed horizontal line represents the FDR threshold of 0.05, and the vertical dashed lines represent the log2FC thresholds (≥ |0.5|). **d,** Venn diagram illustrating the number of DEGs that are shared in the PGCp VS PGCd, PGCd VS GFDd and 50µM ZED1227 VS mock comparisons. e, Heatmap of the 11 DEGs in 50µM ZED1227 VS mock comparison. Genes ordered according to their log2FC. Z-score of normalized expression is plotted. 50µM ZED1227 (n = 3), mock-treated (n = 3).

#List of CEC-3 Trial Group Collaborators

Investigators

**Country**	**Principal Investigator**	**Institution**
Estonia	**Karin Kull, MD**	Department of Gastroenterology, Internal Medicine Clinic, Tartu University Hospital, Tartu
Finland	**Jari Koskenpato, MD**	Lääkärikeskus Aava Helsinki Kamppi, Helsinki
	**Mika Scheinin, MD, PhD**	Clinical Research Services Turku - CRST Oy, Turku
	**Marja-Leena Lähdeaho, MD, PhD***	Faculty of Medicine and Health Technology, Tampere University and Tampere University Hospital, Tampere<br>*and Department of Pediatrics, Tampere University Hospital, Tampere
Germany		
	**Michael Schumann, MD**	Department for Gastroenterology, Infectious diseases and Rheumatology, Campus Benjamin Franklin, Charité - University Medicine Berlin, Berlin
	**Yurdagül Zopf, MD**	Department of Medicine 1, Hector Center for Nutrition, Exercise, and Sports, Universitätsklinikum Erlangen, Friedrich-Alexander-University Erlangen-Nürnberg, Erlangen
	**Andreas Stallmach, MD**	Department of Internal Medicine IV, Jena University Hospital, Friedrich-Schiller University Jena, Jena
	**Ansgar W. Lohse, MD**	I. Department of Medicine, University Medical Center Hamburg-Eppendorf, Hamburg
	**Stefano Fusco, MD**	Division of Gastroenterology, Hepatology, Infectious Diseases, Department of Internal Medicine I, University Hospital Tübingen, Tübingen
Germany	**Jost Langhorst, MD**	Department for Internal and Integrative Medicine, Kliniken Essen-Mitte, Essen
	**Jost Langhorst, MD**	Department for Internal and Integrative Medicine, Sozialstiftung Bamberg, Chair for Integrative Medicine, University of Duisburg-Essen, Bamberg
	**Helga Paula Török, MD**	Department of Medicine II, University Hospital, LMU Munich, Munich
Ireland	**Valerie Byrnes, MD**	University College Hospital Galway, Galway

Country	Principal Investigator	Institution
Lithuania	**Juozas Kupcinskas, MD**	Gastroenterology Department and Institute for Digestive Research, Lithuanian University of Health Sciences, Kaunas
Norway		
	**Øistein Hovde, MD, PhD**	Medical Department, Innlandet Hospital Trust, Gjøvik
	**Jørgen Jahnsen, MD**	Akershus University Hospital, Lørenskog
Switzerland	**Luc Biedermann, MD**	Department of Gastroenterology and Hepatology, University Hospital Zürich, Zürich
	**Jonas Zeitz, MD**	Swiss Celiac Center, Center of Gastroenterology, Clinic Hirslanden, Zürich and Department of Gastroenterology and Hepatology, University Hospital Zürich, Zürich
