# Supplementary material for: Transcriptomic analysis of intestine following administration of a transglutaminase 2 inhibitor to prevent gluten-induced intestinal damage in celiac disease
Source: Nat Immunol. 2024 Jun 24;25(7):1218–30. doi: 10.1038/s41590-024-01867-0 (PMC11224021; doi:10.1038/s41590-024-01867-0)
Supplement: Supplementary file 2 — Reporting Summary [file 41590_2024_1867_MOESM2_ESM.pdf]

nature portfolio

Corresponding author(s): Keijo Viiri

Last updated by author(s): May 7, 2024

Reporting Summary

Nature Portfolio wishes to improve the reproducibility of the work that we publish. This form provides structure for consistency and transparency in reporting. For further information on Nature Portfolio policies, see our Editorial Policies and the Editorial Policy Checklist.

Statistics

For all statistical analyses, confirm that the following items are present in the figure legend, table legend, main text, or Methods section.

n/a	Confirmed	
☐	☒	The exact sample size (*n*) for each experimental group/condition, given as a discrete number and unit of measurement
☒	☐	A statement on whether measurements were taken from distinct samples or whether the same sample was measured repeatedly
☐	☒	The statistical test(s) used AND whether they are one- or two-sided<br>*Only common tests should be described solely by name; describe more complex techniques in the Methods section.*
☐	☒	A description of all covariates tested
☐	☒	A description of any assumptions or corrections, such as tests of normality and adjustment for multiple comparisons
☐	☒	A full description of the statistical parameters including central tendency (e.g. means) or other basic estimates (e.g. regression coefficient) AND variation (e.g. standard deviation) or associated estimates of uncertainty (e.g. confidence intervals)
☐	☒	For null hypothesis testing, the test statistic (e.g. *F*, *t*, *r*) with confidence intervals, effect sizes, degrees of freedom and *P* value noted<br>*Give P values as exact values whenever suitable.*
☒	☐	For Bayesian analysis, information on the choice of priors and Markov chain Monte Carlo settings
☒	☐	For hierarchical and complex designs, identification of the appropriate level for tests and full reporting of outcomes
☐	☒	Estimates of effect sizes (e.g. Cohen's *d*, Pearson's *r*), indicating how they were calculated

Our web collection on statistics for biologists contains articles on many of the points above.

Software and code

Policy information about availability of computer code

Data collection	Duodenal forceps biopsies were collected during clinical drug trial at clinical sites and immersed in PAXgene fixative and processed for paraffin block embedding using a standard formalin-free paraffin-infiltration protocol. Total RNA was extracted from the PaxFPE biopsy specimens using an RNeasy Kit (Qiagen, Hilden, Germany) according to the manufacturer's instructions. Library preparation and transcriptomic data generation was performed by Qiagen NGS Service (Qiagen, Hilden, Germany). Raw data was de-multiplexed and FASTQ files for each sample were generated using the bcl2fastq2 software version 2.20 (Illumina inc.).<br>RNA from duodenal organoids was isolated using RNeasy Kit (Qiagen, Hilden, Germany) by following manufacturer's instructions. Preparation of RNA library and transcriptome sequencing was conducted by Novogene Co., LTD (Cambridge, UK).<br>Data quality was checked using the FastQC version v0.11.9. (Cambridge, UK). 3' adapter sequences were trimmed, reads without adapters were kept and reads with <15 bp were removed. Reads were aligned to the human GRCh38 genome using splice aware aligner STAR version 2.7.6 (New York, US)
Data analysis	R version 4.3.0 (R Foundation for Statistical Computing, Vienna, Austria). Code is deposited at (https://github.com/IntestinalSignallingAndEpigeneticsLab/Dotsenko-et-al.-2024).

For manuscripts utilizing custom algorithms or software that are central to the research but not yet described in published literature, software must be made available to editors and reviewers. We strongly encourage code deposition in a community repository (e.g. GitHub). See the Nature Portfolio guidelines for submitting code & software for further information.

Data

Policy information about availability of data

All manuscripts must include a data availability statement. This statement should provide the following information, where applicable:

- Accession codes, unique identifiers, or web links for publicly available datasets
- A description of any restrictions on data availability
- For clinical datasets or third party data, please ensure that the statement adheres to our policy

Bulk RNA-sequencing data from patient biopsies and patient-derived intestinal organoids described in this study are available in the European Genome-phenome
Bulk RNA-sequencing data from patient biopsies and patient-derived intestinal organoids described in this study are available in the European Genome-phenome
Archive (EGA) under accession number EGAS0000000324. Additional data used in this paper includes Full single cell RNA-seq dataset intestinal regions of adult donors (https://www.gutcellatlas.org/), lists of human duodenal cell types and transporter genes expressed along the upper gastrointestinal tract downloaded from supplementary files included with Busslinger et al. paper (https://www.sciencedirect.com/science/article/pii/S2211124721001339), lists of immune cells marker genes downloaded from supplementary files included with in Atlasy et al., study (https://www.nature.com/articles/s41467-022-32691-5), pathways gene sets used in study downloaded from Human MSigDB Collections at https://www.gsea-msigdb.org/gsea/msigdb/collections.jsp.

Research involving human participants, their data, or biological material

Policy information about studies with human participants or human data. See also policy information about sex, gender (identity/presentation), and sexual orientation and race, ethnicity and racism.

Reporting on sex and gender	The results of the celiac disease clinical drug trial, using the investigational medical product ZED1227, a TG2 inhibitor, is fully reported with supplemental data in Schuppan and Mäki et al., NEJM 2021;385:35-45. In the clinical study sex and gender are taken care of, we attach the original signed trial protocol (confidential, approved by Dr. Falk Pharma). In the present study, we report the results of our celiac expression profiling samples, the gluten challenge study subject biopsy RNASeq results, Zed1227 100 mg vs. placebo drug. Our RNASeq results are here not given separately for males and females, in drug vs placebo (see Extended Data Table 1, patient characteristics as to female %), as we have no indication in the celiac disease literature that gluten-dependent gene transcript up or downregulations at the duodenal mucosal level could be different in males and females.
Reporting on race, ethnicity, or other socially relevant groupings	All Caucasians, no socially relevant groupings were made
Population characteristics	Characteristics of the population used in this study is described in the Extended Data Table 1 "Patient characteristics"
Recruitment	The published drug trial participant recruitment is not copied to this manuscript, we see it is not relevant to repeat it, we cite the NEJM study.
Ethics oversight	We conducted the clinical trial published in NEJM (above) at 20 sites in seven countries (Estonia, Finland, Germany, Ireland, Lithuania, Norway, and Switzerland). The trial was approved by an independent ethics committee at each site. Written informed consent was obtained from each patient before screening. Ethics approvals and informed consents included the pre-specified optional biopsy samples centralized to Tampere for further academic studies (mRNA), the present study. In Finland the protocol was approved by TUKIJA dnro 223/06.00.01/2017, EudraCT 2017-002241-30. For human organoid cultures the protocol was approved by the Ethics Committee of the Tampere University Hospital, Tampere, Finland (ETL-code R18082).

Note that full information on the approval of the study protocol must also be provided in the manuscript.

Field-specific reporting

Please select the one below that is the best fit for your research. If you are not sure, read the appropriate sections before making your selection.

☒ Life sciences ☐ Behavioural & social sciences ☐ Ecological, evolutionary & environmental sciences

For a reference copy of the document with all sections, see nature.com/documents/nr-reporting-summary-flat.pdf

Life sciences study design

All studies must disclose on these points even when the disclosure is negative.

Sample size	Sample size was not predetermined statistically. Participant recruitment and sample collection was done in clinical trial reported in Schuppan and Mäki et al., NEJM 2021;385:35-45.<br>For the current study, we used all available samples from ZED1227 100 mg and placebo arms of the trial.
Data exclusions	Exclusion criteria were not pre-established. One sample was excluded from secondary differential expression analysis, as it had low total reads. The mean of Total reads for all the samples is 3.51 ± 0.07 million reads. Excluded sample achieved only 1.13 million reads. That sample

	is excluded from analyses and all subsequent calculations are performed on 115 left samples.
Replication	Findings of this study are restricted to the studied cohorts and replication studies were not possible to include. We obtained RNA extracted from PaxFPE biopsy specimens from one single randomized double-blind placebo-controlled clinical drug trial, CEC-3/CEL, conducted from May 16, 2018, to February 27, 2020 and published in NEJM 2021 by Schuppan & Mäki et al.
Randomization	Data collection and randomization was performed prior to current study as described in Schuppan and Mäki et al., NEJM 2021;385:35-45.
Blinding	Schuppan & Mäki et al. drug trial published in NEJM 2021 was a randomized double-blind gluten challenge trial. For the present study we received the biopsies of the placebo and the ZED1227 100 mg drug arms. All RNA-Seq studies were run in parallel and at the same time. To be able to give results as to baseline and post gluten challenge in placebo vs. drug arm, the trial sponsor provided us with the codes for blinding.

Behavioural & social sciences study design

All studies must disclose on these points even when the disclosure is negative.

Study description	*Briefly describe the study type including whether data are quantitative, qualitative, or mixed-methods (e.g. qualitative cross-sectional, quantitative experimental, mixed-methods case study).*
Research sample	*State the research sample (e.g. Harvard university undergraduates, villagers in rural India) and provide relevant demographic information (e.g. age, sex) and indicate whether the sample is representative. Provide a rationale for the study sample chosen. For studies involving existing datasets, please describe the dataset and source.*
Sampling strategy	*Describe the sampling procedure (e.g. random, snowball, stratified, convenience). Describe the statistical methods that were used to predetermine sample size OR if no sample-size calculation was performed, describe how sample sizes were chosen and provide a rationale for why these sample sizes are sufficient. For qualitative data, please indicate whether data saturation was considered, and what criteria were used to decide that no further sampling was needed.*
Data collection	*Provide details about the data collection procedure, including the instruments or devices used to record the data (e.g. pen and paper, computer, eye tracker, video or audio equipment) whether anyone was present besides the participant(s) and the researcher, and whether the researcher was blind to experimental condition and/or the study hypothesis during data collection.*
Timing	*Indicate the start and stop dates of data collection. If there is a gap between collection periods, state the dates for each sample cohort.*
Data exclusions	*If no data were excluded from the analyses, state so OR if data were excluded, provide the exact number of exclusions and the rationale behind them, indicating whether exclusion criteria were pre-established.*
Non-participation	*State how many participants dropped out/declined participation and the reason(s) given OR provide response rate OR state that no participants dropped out/declined participation.*
Randomization	*If participants were not allocated into experimental groups, state so OR describe how participants were allocated to groups, and if allocation was not random, describe how covariates were controlled.*

Ecological, evolutionary & environmental sciences study design

All studies must disclose on these points even when the disclosure is negative.

Study description	*Briefly describe the study. For quantitative data include treatment factors and interactions, design structure (e.g. factorial, nested, hierarchical), nature and number of experimental units and replicates.*
Research sample	*Describe the research sample (e.g. a group of tagged Passer domesticus, all Stenocereus thurberi within Organ Pipe Cactus National Monument), and provide a rationale for the sample choice. When relevant, describe the organism taxa, source, sex, age range and any manipulations. State what population the sample is meant to represent when applicable. For studies involving existing datasets, describe the data and its source.*
Sampling strategy	*Note the sampling procedure. Describe the statistical methods that were used to predetermine sample size OR if no sample-size calculation was performed, describe how sample sizes were chosen and provide a rationale for why these sample sizes are sufficient.*
Data collection	*Describe the data collection procedure, including who recorded the data and how.*
Timing and spatial scale	*Indicate the start and stop dates of data collection, noting the frequency and periodicity of sampling and providing a rationale for these choices. If there is a gap between collection periods, state the dates for each sample cohort. Specify the spatial scale from which the data are taken*
Data exclusions	*If no data were excluded from the analyses, state so OR if data were excluded, describe the exclusions and the rationale behind them, indicating whether exclusion criteria were pre-established.*
Reproducibility	*Describe the measures taken to verify the reproducibility of experimental findings. For each experiment, note whether any attempts to repeat the experiment failed OR state that all attempts to repeat the experiment were successful.*

Randomization	*Describe how samples/organisms/participants were allocated into groups. If allocation was not random, describe how covariates were controlled. If this is not relevant to your study, explain why.*
Blinding	*Describe the extent of blinding used during data acquisition and analysis. If blinding was not possible, describe why OR explain why blinding was not relevant to your study.*

Did the study involve field work? ☐ Yes ☐ No

Field work, collection and transport

Field conditions	*Describe the study conditions for field work, providing relevant parameters (e.g. temperature, rainfall).*
Location	*State the location of the sampling or experiment, providing relevant parameters (e.g. latitude and longitude, elevation, water depth).*
Access & import/export	*Describe the efforts you have made to access habitats and to collect and import/export your samples in a responsible manner and in compliance with local, national and international laws, noting any permits that were obtained (give the name of the issuing authority, the date of issue, and any identifying information).*
Disturbance	*Describe any disturbance caused by the study and how it was minimized.*

Reporting for specific materials, systems and methods

We require information from authors about some types of materials, experimental systems and methods used in many studies. Here, indicate whether each material, system or method listed is relevant to your study. If you are not sure if a list item applies to your research, read the appropriate section before selecting a response.

Materials & experimental systems

n/a	Involved in the study	
☐	☐	Antibodies
☐	☒	Eukaryotic cell lines
☐	☐	Palaeontology and archaeology
☐	☐	Animals and other organisms
☐	☒	Clinical data
☐	☐	Dual use research of concern
☐	☐	Plants

Methods

n/a	Involved in the study	
☐	☐	ChIP-seq
☐	☐	Flow cytometry
☐	☐	MRI-based neuroimaging

Antibodies

Antibodies used	*Describe all antibodies used in the study; as applicable, provide supplier name, catalog number, clone name, and lot number.*
Validation	*Describe the validation of each primary antibody for the species and application, noting any validation statements on the manufacturer's website, relevant citations, antibody profiles in online databases, or data provided in the manuscript.*

Eukaryotic cell lines

Policy information about cell lines and Sex and Gender in Research

Cell line source(s)	Caco-2 cells (ATCC, Manassas, USA)
Authentication	none
Mycoplasma contamination	negative
Commonly misidentified lines (See ICLAC register)	No commonly misidentified cell lines were used

Palaeontology and Archaeology

Specimen provenance	*Provide provenance information for specimens and describe permits that were obtained for the work (including the name of the issuing authority, the date of issue, and any identifying information). Permits should encompass collection and, where applicable,*

	export.
Specimen deposition	*Indicate where the specimens have been deposited to permit free access by other researchers.*
Dating methods	*If new dates are provided, describe how they were obtained (e.g. collection, storage, sample pretreatment and measurement), where they were obtained (i.e. lab name), the calibration program and the protocol for quality assurance OR state that no new dates are provided.*

☐ Tick this box to confirm that the raw and calibrated dates are available in the paper or in Supplementary Information.

Ethics oversight	*Identify the organization(s) that approved or provided guidance on the study protocol, OR state that no ethical approval or guidance was required and explain why not.*

Note that full information on the approval of the study protocol must also be provided in the manuscript.

Animals and other research organisms

Policy information about studies involving animals; ARRIVE guidelines recommended for reporting animal research, and Sex and Gender in Research

Laboratory animals	*For laboratory animals, report species, strain and age OR state that the study did not involve laboratory animals.*
Wild animals	*Provide details on animals observed in or captured in the field; report species and age where possible. Describe how animals were caught and transported and what happened to captive animals after the study (if killed, explain why and describe method; if released, say where and when) OR state that the study did not involve wild animals.*
Reporting on sex	*Indicate if findings apply to only one sex; describe whether sex was considered in study design, methods used for assigning sex. Provide data disaggregated for sex where this information has been collected in the source data as appropriate; provide overall numbers in this Reporting Summary. Please state if this information has not been collected. Report sex-based analyses where performed, justify reasons for lack of sex-based analysis.*
Field-collected samples	*For laboratory work with field-collected samples, describe all relevant parameters such as housing, maintenance, temperature, photoperiod and end-of-experiment protocol OR state that the study did not involve samples collected from the field.*
Ethics oversight	*Identify the organization(s) that approved or provided guidance on the study protocol, OR state that no ethical approval or guidance was required and explain why not.*

Note that full information on the approval of the study protocol must also be provided in the manuscript.

Clinical data

Policy information about clinical studies
All manuscripts should comply with the ICMJE guidelines for publication of clinical research and a completed CONSORT checklist must be included with all submissions.

Clinical trial registration	The published clinical drug trial registration is EudraCT 2017-002241-30 (see NEJM 2021;385:35-45).
Study protocol	The signed drug trial final protocol is attached (confidential, approved by Dr. Falk Pharma)
Data collection	As published in NEJM, our European multicenter trial was conducted from May 16, 2018, to February 27, 2020 and data was collected according to the protocol and the biopsy samples for the present study were centralized to Tampere university where we performed the pre-specified mRNA studies. The randomized, double-blind, and placebo-controlled clinical drug trial CEC-3/CEL, EudraCT No.: 2017-002241-30, published as Schuppan and Mäki et al. in New England Journal of Medicine, 2021;385:35-45, was conducted in 20 sites in 7 countries, at university and public/private hospitals and trial center settings: Estonia (Tartu University Hospital, Tartu), Finland (Aava Kamppi Medical Centre, Helsinki; Clinical Research Services Turku – CRST Oy, Turku; FinnMedi Oy, Clinical Trial Center, Tampere), Germany (Institute of Translational Immunology, University Medical Center of the Johannes Gutenberg University, Mainz; Charité University Medicine Berlin, Campus Benjamin Franklin (CBF), Berlin; University Hospital Erlangen, Erlangen; University Hospital of Jena, Jena; University Medical Center Hamburg-Eppendorf, 1st Department of Medicine, Hamburg; Medical University Hospital Tübingen, Internal Medicine 1, Tübingen; Protestant Hospital Essen Steele, Clinic for Naturopathy and Integrative Medicine, Essen; Hospital of the University of Munich-Grosshadern, Medical Clinic and Out-Patient Clinic II, Munich; Clinic for Integrative Medicine and Naturopathy Social Foundation Bamberg "Klinik am Bruderwald", Bamberg), Ireland (University College Hospital Galway, HRB Clinical Research Facility, National University of Ireland, Galway), Lithuania (Hospital of Lithuanian University of Health Sciences, Kauno klinikos, Department of Gastroenterology, Kaunas), Norway (Oslo University Hospital – Rikshospitalet, Oslo; Gjøvik Hospital, Gjøvik; Akershus University Hospital, Lørenskog), and Switzerland (University Hospital Zurich, Department of Gastroenterology and Hepatology, Zürich; Center of Gastroenterology, The Hirslanden Private Clinic Group, Zürich). One site in Austria was initiated but did not enroll any subjects. Chemical laboratory and biopsy samples were centralized for processings and readings and biopsy-extracted RNA was shipped for present studies to us at Tampere University, Faculty of Medicine and Health Technology, Tampere, Finland.
Outcomes	The pre-defined primary and secondary outcome measures of the trial is to be found in the NEJM publication and attached study protocol. We included in the study protocol certain exploratory outcomes to avoid repeating this kind of hugh randomized clinical trial just to get placebo and drug arm biopsy samples during a gluten challenge. It was possible to incorporate this kind of academic study to the industry-sponsored drug trial. Thus, we were able to perform a genome-wide RNASeq on already collected prospective and pre-specified samples. The protocol page 44 says that the biopsies will be used for further immunohistochemistry (IHC) and/or

messenger ribonucleic acid (mRNA) analyses depending on the clinical outcome of the trial (optional investigation). As the clinical outcome published in NEJM was excellent, the trial sponsor, Dr. Falk Pharma, made an agreement with Tampere university and allowed us to proceed with the pre-specified exploratory outcomes/optional samples, and we performed the present study.

Dual use research of concern

Policy information about dual use research of concern

Hazards

Could the accidental, deliberate or reckless misuse of agents or technologies generated in the work, or the application of information presented in the manuscript, pose a threat to:

No	Yes	
☒	☐	Public health
☒	☐	National security
☒	☐	Crops and/or livestock
☒	☐	Ecosystems
☒	☐	Any other significant area

Experiments of concern

Does the work involve any of these experiments of concern:

No	Yes	
☒	☐	Demonstrate how to render a vaccine ineffective
☒	☐	Confer resistance to therapeutically useful antibiotics or antiviral agents
☒	☐	Enhance the virulence of a pathogen or render a nonpathogen virulent
☒	☐	Increase transmissibility of a pathogen
☒	☐	Alter the host range of a pathogen
☒	☐	Enable evasion of diagnostic/detection modalities
☒	☐	Enable the weaponization of a biological agent or toxin
☒	☐	Any other potentially harmful combination of experiments and agents

Plants

Seed stocks

Report on the source of all seed stocks or other plant material used. If applicable, state the seed stock centre and catalogue number. If plant specimens were collected from the field, describe the collection location, date and sampling procedures.

Novel plant genotypes

Describe the methods by which all novel plant genotypes were produced. This includes those generated by transgenic approaches, gene editing, chemical/radiation-based mutagenesis and hybridization. For transgenic lines, describe the transformation method, the number of independent lines analyzed and the generation upon which experiments were performed. For gene-edited lines, describe the editor used, the endogenous sequence targeted for editing, the targeting guide RNA sequence (if applicable) and how the editor was applied.

Authentication

Describe any authentication procedures for each seed stock used or novel genotype generated. Describe any experiments used to assess the effect of a mutation and, where applicable, how potential secondary effects (e.g. second site T-DNA insertions, mosiacism, off-target gene editing) were examined.

ChIP-seq

Data deposition

☐ Confirm that both raw and final processed data have been deposited in a public database such as GEO.

☐ Confirm that you have deposited or provided access to graph files (e.g. BED files) for the called peaks.

Data access links
May remain private before publication.

For "Initial submission" or "Revised version" documents, provide reviewer access links. For your "Final submission" document, provide a link to the deposited data.

Files in database submission

Provide a list of all files available in the database submission.

Genome browser session
(e.g. UCSC)

Provide a link to an anonymized genome browser session for "Initial submission" and "Revised version" documents only, to enable peer review. Write "no longer applicable" for "Final submission" documents.

Methodology

Replicates

Describe the experimental replicates, specifying number, type and replicate agreement.

Sequencing depth

Describe the sequencing depth for each experiment, providing the total number of reads, uniquely mapped reads, length of reads and whether they were paired- or single-end.

Antibodies

Describe the antibodies used for the ChIP-seq experiments; as applicable, provide supplier name, catalog number, clone name, and lot number.

Peak calling parameters

Specify the command line program and parameters used for read mapping and peak calling, including the ChIP, control and index files used.

Data quality

Describe the methods used to ensure data quality in full detail, including how many peaks are at FDR 5% and above 5-fold enrichment.

Software

Describe the software used to collect and analyze the ChIP-seq data. For custom code that has been deposited into a community repository, provide accession details.

Flow Cytometry

Plots

Confirm that:

- [ ] The axis labels state the marker and fluorochrome used (e.g. CD4-FITC).
- [ ] The axis scales are clearly visible. Include numbers along axes only for bottom left plot of group (a 'group' is an analysis of identical markers).
- [ ] All plots are contour plots with outliers or pseudocolor plots.
- [ ] A numerical value for number of cells or percentage (with statistics) is provided.

Methodology

Sample preparation

Describe the sample preparation, detailing the biological source of the cells and any tissue processing steps used.

Instrument

Identify the instrument used for data collection, specifying make and model number.

Software

Describe the software used to collect and analyze the flow cytometry data. For custom code that has been deposited into a community repository, provide accession details.

Cell population abundance

Describe the abundance of the relevant cell populations within post-sort fractions, providing details on the purity of the samples and how it was determined.

Gating strategy

Describe the gating strategy used for all relevant experiments, specifying the preliminary FSC/SSC gates of the starting cell population, indicating where boundaries between "positive" and "negative" staining cell populations are defined.

- [ ] Tick this box to confirm that a figure exemplifying the gating strategy is provided in the Supplementary Information.

Magnetic resonance imaging

Experimental design

Design type

Indicate task or resting state; event-related or block design.

Design specifications

Specify the number of blocks, trials or experimental units per session and/or subject, and specify the length of each trial or block (if trials are blocked) and interval between trials.

Behavioral performance measures

State number and/or type of variables recorded (e.g. correct button press, response time) and what statistics were used to establish that the subjects were performing the task as expected (e.g. mean, range, and/or standard deviation across subjects).

Acquisition

Imaging type(s) — *Specify: functional, structural, diffusion, perfusion.*

Field strength — *Specify in Tesla*

Sequence & imaging parameters — *Specify the pulse sequence type (gradient echo, spin echo, etc.), imaging type (EPI, spiral, etc.), field of view, matrix size, slice thickness, orientation and TE/TR/flip angle.*

Area of acquisition — *State whether a whole brain scan was used OR define the area of acquisition, describing how the region was determined.*

Diffusion MRI ☐ Used ☐ Not used

Preprocessing

Preprocessing software — *Provide detail on software version and revision number and on specific parameters (model/functions, brain extraction, segmentation, smoothing kernel size, etc.).*

Normalization — *If data were normalized/standardized, describe the approach(es): specify linear or non-linear and define image types used for transformation OR indicate that data were not normalized and explain rationale for lack of normalization.*

Normalization template — *Describe the template used for normalization/transformation, specifying subject space or group standardized space (e.g. original Talairach, MNI305, ICBM152) OR indicate that the data were not normalized.*

Noise and artifact removal — *Describe your procedure(s) for artifact and structured noise removal, specifying motion parameters, tissue signals and physiological signals (heart rate, respiration).*

Volume censoring — *Define your software and/or method and criteria for volume censoring, and state the extent of such censoring.*

Statistical modeling & inference

Model type and settings — *Specify type (mass univariate, multivariate, RSA, predictive, etc.) and describe essential details of the model at the first and second levels (e.g. fixed, random or mixed effects; drift or auto-correlation).*

Effect(s) tested — *Define precise effect in terms of the task or stimulus conditions instead of psychological concepts and indicate whether ANOVA or factorial designs were used.*

Specify type of analysis: ☐ Whole brain ☐ ROI-based ☐ Both

Statistic type for inference — *Specify voxel-wise or cluster-wise and report all relevant parameters for cluster-wise methods.*

(See Eklund et al. 2016)

Correction — *Describe the type of correction and how it is obtained for multiple comparisons (e.g. FWE, FDR, permutation or Monte Carlo).*

Models & analysis

n/a	Involved in the study
☐	☐ Functional and/or effective connectivity
☐	☐ Graph analysis
☐	☐ Multivariate modeling or predictive analysis

Functional and/or effective connectivity — *Report the measures of dependence used and the model details (e.g. Pearson correlation, partial correlation, mutual information).*

Graph analysis — *Report the dependent variable and connectivity measure, specifying weighted graph or binarized graph, subject- or group-level, and the global and/or node summaries used (e.g. clustering coefficient, efficiency, etc.).*

Multivariate modeling and predictive analysis — *Specify independent variables, features extraction and dimension reduction, model, training and evaluation metrics.*
