# Supplementary material for: Transcriptomic analysis of intestine following administration of a transglutaminase 2 inhibitor to prevent gluten-induced intestinal damage in celiac disease
Source: Nat Immunol. 2024 Jun 24;25(7):1218–30. doi: 10.1038/s41590-024-01867-0 (PMC11224021; doi:10.1038/s41590-024-01867-0)
Supplement: Supplementary file 9 — Source data for Supplementary Tables 1 and 2 and Figs. 1–5. [file 41590_2024_1867_MOESM9_ESM.pdf]

Summary

1. Data used in Figure 4 and Supplemental figure S6

2. Repeated measures ANOVA (figure 4A)

3. Two-way ANCOVA (figure 4B)

4. One-way ANCOVA (figure 4C)

5. Two-way ANCOVA (figure 4D)

6. One-way ANCOVA (figure S4A)

7. One-way ANCOVA (figure S4B)

8. Session information.

Supplementary Information for Figure 4: The effects of HLA-DQ genetic background on VH:CrD and gene expression

Summary

We assessed the impact of treatment on VH:CrD within different timepoints (GFD and PGC) across HLA-DQ genetic background groups (G1, G2, and G3) by fitting repeated measures ANOVA.

In the placebo group, the interaction between timepoint and HLA-DQ genetic groups was statistically significant F(2,20) = 7.9, *P* = .003 (2.3 Computation of Repeated measures ANOVA), indicating that HLA-DQ genetic background has impact on VH:CrD in a certain timepoint. Indeed, the one-way model suggests that the simple main effect of HLA-DQ genetic groups was not significant at the GFDp time point (*P*.adj = 0.484), but it is significant at PGCp (*P*.adj = 0.042) (2.4.1 Simple main effect of Genotype group). Pairwise comparisons between timepoints for placebo group are presented 2.4.3 Simple pairwise comparisons..

The interaction term in the drug group was not significant (F (2,31) = 3.02, P = .06) (2.3 Computation of Repeated measures ANOVA). The main effect of time (GFDd vs. PGCd) on VH:CrD was statistically significant (*P* =.005) based on the comparison between these two groups consisting of 34 subjects each 2.4.2 Main effects for each of the two variables: Treatment and HLA_Genotype_Group. The main effect of different genotype groups (G1, G2, and G3) on VH:CrD for at GFDd and PGCd time points was assessed by pairwise comparisons. These p-values suggest that the impact of HLA-DQ genetic background may be statistically significant for the G1 group (*P* = .047), but not significant for the G2 (*P* = .069) and G3 groups (*P* = .389) 2.4.3 Simple pairwise comparisons..

When examining the changes in mean VH:CrD within genotype groups over time (2.4.4 Comparisons plot (Figure 4A).), it is evident that the groups exhibit varying trajectories of change. Notably, the slope of the G1 group appears to deviate the most from the parallel pattern among the groups for both drug and placebo treatments.

Given the notable drop in the VH:CrD after ZED1227 treatment in high gluten-response genotype group G1, we analyzed the efficacy of treatments in each genotype group. A two-way analysis of covariance (ANCOVA) statistical analysis was performed to examine the effects of treatment and HLA-DQ genetic background on VH:CrD at PGC. After adjustment for the VH:CrD at GFD, there was no statistically significant interaction between treatment and the HLA-DQ genotype group on the histomorphometry parameters (F (2,50) = 2.2, P = .12) 3.3 Computation of two-way ANCOVA. Pairwise multiple comparisons show significant difference between the PGC VH:CrD means adjusted for GFD in all genotype groups between drug and placebo patients (3.4.2. Pairwise comparisons plot). This suggests that, although the G1 group patients had a significant VH:CrD decrease after gluten challenge in the drug group, the VH:CrD ratio was still significantly higher in the drug group compared to the placebo group, irrespective of the genotype.

A one-way ANCOVA statistical analysis was performed to further examine the significantly weaker recovery of VH:CrD with ZED1227 in the genotype G1 group. The data showed that there was an effect of HLA-DQ genetic background on the VH:CrD value at PGCd adjusted for VH:CrD at GFDd values (F(2, 30) = 5.11, P = .012) 4.3 Computation of one-way ANCOVA. The estimated difference in the VH:CrD ratio for drug patients belonging to G3 genotypes versus G1 genotypes is -0.52 (95% CI -0.86 to -0.19) with P adj = .01. Other estimated differences (G3-G2 and G2-G1) were not significant but showed the tendency of group G2 having the intermediate position between G1 and G3, when judging by the VH:CrD value (4.4.2. Pairwise comparisons plot).

Interestingly, the G1 high-risk genotype specifically affected the villous height (6.4.2. Pairwise comparisons plot) and not crypt depth (7.4.2. Pairwise comparisons plot).

The CeD pathophysiological epithelial IFN-γ response was again studied with a two-way ANCOVA statistical analysis F (2,49) = 0,071, *P* = .93 5.3 Computation of two-way ANCOVA , and pairwise comparisons showed that PGCd patients in the G1 genotype group still had IFN-γ response active and did not statistically differ from the placebo group (5.4.3. Pairwise comparisons plot).

1. Data used in Figure 4 and Supplemental figure S6

Data used in figure 4 and supplemental figure S6. VH:Crd - villus height to crypt depth ratio

Show 10 entries Search:

Table.1 Data used in Figure 4 and Supplemental figure S6

	Patient_ID	treatment	timepoint	HLA_Genotype_Group	CrD	VH	VHCrD	epithelial_IFNg_responce
1	1	placebo	GFD	G2	222.04	352.62	1.59	-1.1
2	1	placebo	PGC	G2	350.81	169.59	0.48	1.14
3	2	drug	GFD	G1	212.62	344.41	1.62	-0.77
4	2	drug	PGC	G1	221.56	295.07	1.33	0.33
5	3	drug	GFD	G1	183.68	394.64	2.15	0.45
6	3	drug	PGC	G1	213.07	299.37	1.41	3.67
7	4	drug	GFD	G2	159.51	359.68	2.25	-0.98
8	4	drug	PGC	G2	199.58	416.09	2.08	-1.27
9	5	drug	GFD	G3	163.29	370.17	2.27	-1.19
10	5	drug	PGC	G3	159.38	405.11	2.54	0.35

Showing 1 to 10 of 116 entries

Previous 1 2 3 4 5 … 12 Next

2. Repeated measures ANOVA (figure 4A)

The objective is to determine whether belonging to a specific HLA-DQ genotype group leads to a significant decrease in VH:CrD over time during gluten challenge. In essence, the aim is to identify if there is a interaction between HLA-DQ genotype group and Timepoint concerning VH:CrD.

2.1 Summary statistics

One patient from placebo group, failed during identification of HLA-DQ genotype. This patient is marked as "not identified" in Table below and was excluded from subsequent analysis.

```
## # A tibble: 14 × 7
##    treatment timepoint HLA_Genotype_Group variable     n  mean    sd
##    <chr>     <chr>     <chr>              <fct>    <dbl> <dbl> <dbl>
##  1 drug      GFD       G1                 VHCrD        6  2.08  0.5 
##  2 drug      PGC       G1                 VHCrD        6  1.53  0.23
##  3 drug      GFD       G2                 VHCrD       14  2.05  0.27
##  4 drug      PGC       G2                 VHCrD       14  1.84  0.46
##  5 drug      GFD       G3                 VHCrD       14  2.17  0.33
##  6 drug      PGC       G3                 VHCrD       14  2.1   0.27
##  7 placebo   GFD       G1                 VHCrD        2  2.23  0.26
##  8 placebo   PGC       G1                 VHCrD        2  0.54  0.15
##  9 placebo   GFD       G2                 VHCrD        6  1.77  0.38
## 10 placebo   PGC       G2                 VHCrD        6  1.08  0.56
## 11 placebo   GFD       G3                 VHCrD       15  2     0.35
## 12 placebo   PGC       G3                 VHCrD       15  1.63  0.57
## 13 placebo   GFD       Not identified     VHCrD        1  1.54 NA   
## 14 placebo   PGC       Not identified     VHCrD        1  0.47 NA   
```

2.2 Assumptions check

2.2.1 Normality assumption

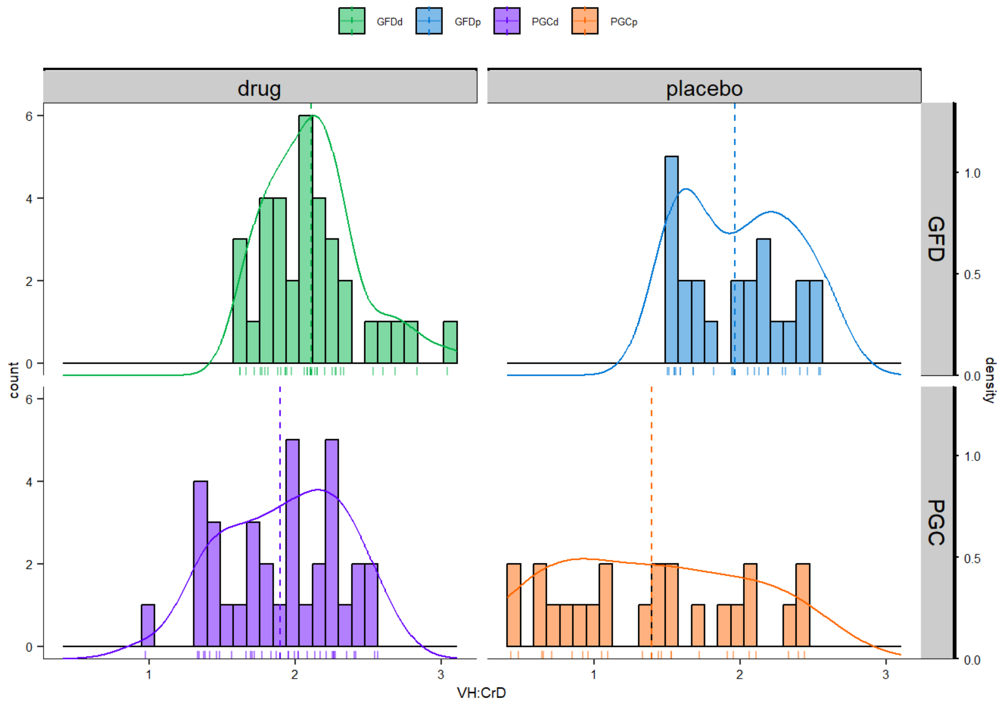

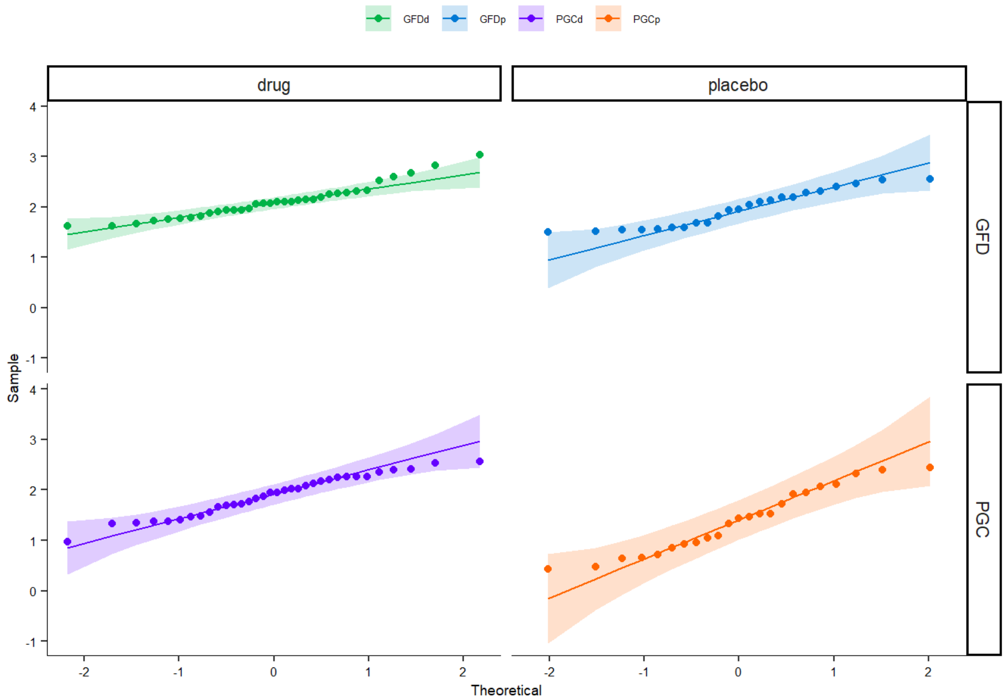

From the plots above, as all the points fall approximately along the reference line, we can assume normality.

2.2.2 Outliers check

```
## # A tibble: 1 × 7
##   Patient_ID treatment timepoint HLA_Genotype_Group VHCrD is.outlier is.extreme
##        <dbl> <chr>     <chr>     <chr>              <dbl> <lgl>      <lgl>
## 1         29 drug      GFD       G1                  3.04 TRUE       FALSE
```

There were no extreme outliers in our data set.

2.3 Computation of Repeated measures ANOVA

Repeated measures ANOVA was employed to assess the impact of treatment on VH:CrD within different timepoints (GFD and PGC) across HLA-DQ genetic background groups (G1, G2, and G3). This analysis comprised 57 patients with identifiable HLA-DQ genotypes. Model included “VH:CrD” as dependent variable, “Genotype group” as between-subject factor variables, “timepoint” as within-subjects factor variables and “Patient ID” as individuals identifier.

```
## # A tibble: 6 × 8
##   treatment Effect                          DFn   DFd     F       p `p<.05`   ges
## * <fct>     <chr>                         <dbl> <dbl> <dbl>   <dbl> <chr>   <dbl>
## 1 drug      HLA_Genotype_Group                2    31  3.06 6.1 e-2 ""      0.121
## 2 drug      timepoint                         1    31 14.7  5.77e-4 "*"     0.126
## 3 drug      HLA_Genotype_Group:timepoint      2    31  3.02 6.3 e-2 ""      0.056
## 4 placebo   HLA_Genotype_Group                2    20  2.52 1.06e-1 ""      0.162
## 5 placebo   timepoint                         1    20 51.8  5.74e-7 "*"     0.377
## 6 placebo   HLA_Genotype_Group:timepoint      2    20  7.94 3   e-3 "*"     0.156
```

The results show the effects of treatment, timepoint, and the interaction between HLA-DQ genetic groups and timepoint for both the drug and placebo groups, along with corresponding degrees of freedom (DFn and DFd), F-values, p-values, significance indicators (*), and effect sizes (ges).

The impact of treatment on VH:CrD at different timepoints (GFD and PGC) was evaluated across HLA-DQ genetic background groups (G1, G2, and G3) using repeated measures ANOVA. In the placebo group, the interaction between timepoint and HLA-DQ genetic groups was statistically significant F(2,20) = 7.9, P = 0.003.

2.4 Post-hoc tests

A significant two-way interaction indicates that the impact that one factor (e.g., HLA_Genotype_Group) has on the outcome variable (e.g., timepoint) depends on the level of the other factor (e.g., timepoint). So, we decompose a significant two-way interaction into:

2.4.1 Simple main effect of Genotype group

Simple main effect of Genotype group on timepoint calculated for ***placebo group***.

```
## # A tibble: 2 × 9
##   timepoint Effect               DFn   DFd     F     p `p<.05`   ges p.adj
##   <fct>     <chr>              <dbl> <dbl> <dbl> <dbl> <chr>   <dbl> <dbl>
## 1 GFD       HLA_Genotype_Group     2    20  1.52 0.242 ""      0.132 0.484
## 2 PGC       HLA_Genotype_Group     2    20  4.72 0.021 "*"     0.32  0.042
```

Considering the Bonferroni adjusted p-value (p.adj), it can be seen that the simple main effect of HLA_Genotype_Group was not significant at the timepoint GFD (p.adj = 0.484). It becomes significant at PGC (p.adj = 0.042).

2.4.2 Main effects for each of the two variables: Treatment and HLA_Genotype_Group

For ***drug group*** the interaction is not significant F(2,31) = 3, P = 0.063, we interpret the main effects for significant timepoint. A significant main effect is followed by pairwise comparisons.

```
## # A tibble: 1 × 10
##   .y.   group1 group2    n1    n2 statistic    df     p p.adj p.adj.signif
## * <chr> <chr>  <chr>  <int> <int>     <dbl> <dbl> <dbl> <dbl> <chr>
## 1 VHCrD GFD    PGC       34    34      3.02    33 0.005 0.005 **
```

The main effect of time (GFDd vs. PGCd) on VH:CrD was statistically significant (P = 0.005) based on the comparison between these two groups consisting of 34 subjects each.

2.4.3 Simple pairwise comparisons.

Multiple pairwise comparisons were performed to determine which groups are different.

```
## # A tibble: 6 × 7
##   treatment HLA_Genotype_Group group1 group2 statistic    df     p
##   <fct>     <chr>              <chr>  <chr>      <dbl> <dbl> <dbl>
## 1 drug      G1                 GFD    PGC        2.62      5 0.047
## 2 drug      G2                 GFD    PGC        1.98     13 0.069
## 3 drug      G3                 GFD    PGC        0.892    13 0.389
## 4 placebo   G1                 GFD    PGC       22.6       1 0.028
## 5 placebo   G2                 GFD    PGC        3.29      5 0.022
## 6 placebo   G3                 GFD    PGC        3.32     14 0.005
```

The main effect of different genotype groups (G1, G2, and G3) on VH:CrD for at GFDd and PGCd time points was assessed by pairwise comparisons. These p-values suggest that the impact of HLA-DQ genetic background may be statistically significant for the G1 group (P = 0.047), but not significant for the G2 (P = 0.069) and G3 groups (P = 0.389).

2.4.4 Comparisons plot (Figure 4A).

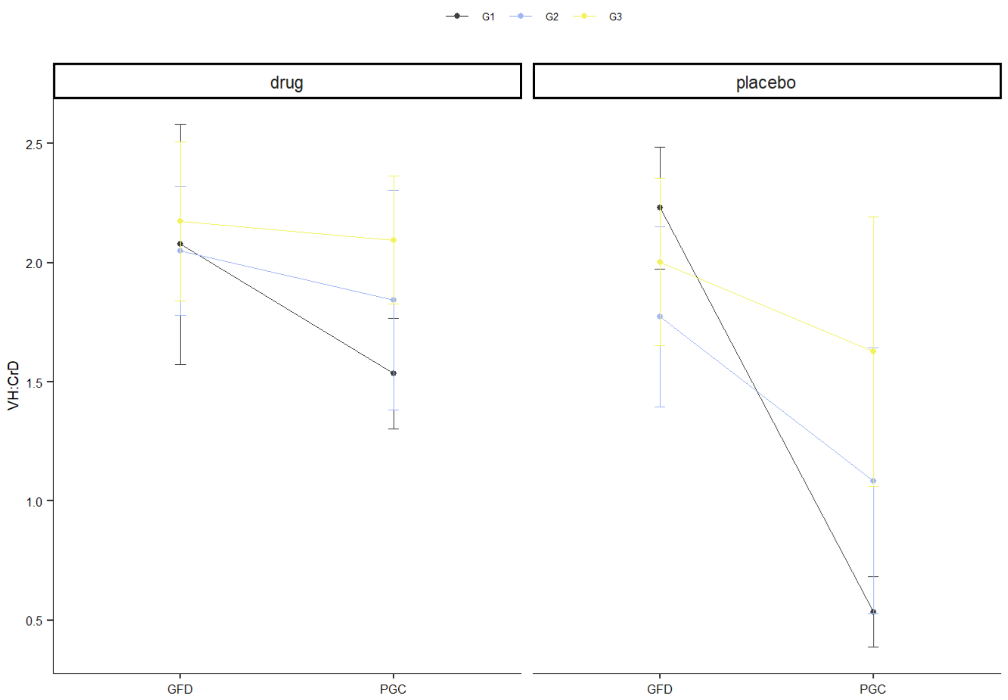

VH:CrD ratio remains higher in the drug group compared to the placebo group, regardless of the genotype. Subjects (n = 57) were divided into two groups according to the treatment received (drug or placebo).

3. Two-way ANCOVA (figure 4B)

Given the notable drop in the VH:CrD after ZED1227 treatment in high gluten-response genotype group G1, we analyzed the efficacy of treatments in each genotype group. A two-way analysis of covariance (ANCOVA) statistical analysis was performed to examine the effects of treatment and HLA-DQ genetic background on VH:CrD at PGC.

To assess the interaction between treatment groups and HLA-DQ genetic backgrounds on VH:CrD at PGC, a two-way ANCOVA was conducted using VH:CrD at PGC as the dependent variable, HLA-DQ genetic background (G1, G2, and G3 genotype groups) and treatment (placebo or drug) as independent variables, and baseline VH:CrD (from GFD group) as a covariate. This analysis included 57 patients, with one subject from the placebo group excluded due to unidentified allele type. The study formulated two null hypotheses for the two-way ANCOVA analysis: 1) no VH:CrD difference at PCG exists between treatment groups (placebo and drug), while accounting for VH:CrD at GFD, and 2) no VH:CrD differences at PCG exist across HLA-DQ genetic backgrounds (G1, G2, and G3 genotype groups), controlling for VH:CrD at GFD.

3.1 Summary statistics

```
## # A tibble: 12 × 7
##    treatment timepoint HLA_Genotype_Group variable     n  mean    sd
##    <fct>     <fct>     <chr>              <fct>    <dbl> <dbl> <dbl>
##  1 drug      GFD       G1                 VHCrD        6  2.08  0.5 
##  2 drug      PGC       G1                 VHCrD        6  1.53  0.23
##  3 drug      GFD       G2                 VHCrD       14  2.05  0.27
##  4 drug      PGC       G2                 VHCrD       14  1.84  0.46
##  5 drug      GFD       G3                 VHCrD       14  2.17  0.33
##  6 drug      PGC       G3                 VHCrD       14  2.1   0.27
##  7 placebo   GFD       G1                 VHCrD        2  2.23  0.26
##  8 placebo   PGC       G1                 VHCrD        2  0.54  0.15
##  9 placebo   GFD       G2                 VHCrD        6  1.77  0.38
## 10 placebo   PGC       G2                 VHCrD        6  1.08  0.56
## 11 placebo   GFD       G3                 VHCrD       15  2     0.35
## 12 placebo   PGC       G3                 VHCrD       15  1.63  0.57
```

3.2 Assumptions check

3.2.1 Normality assumption

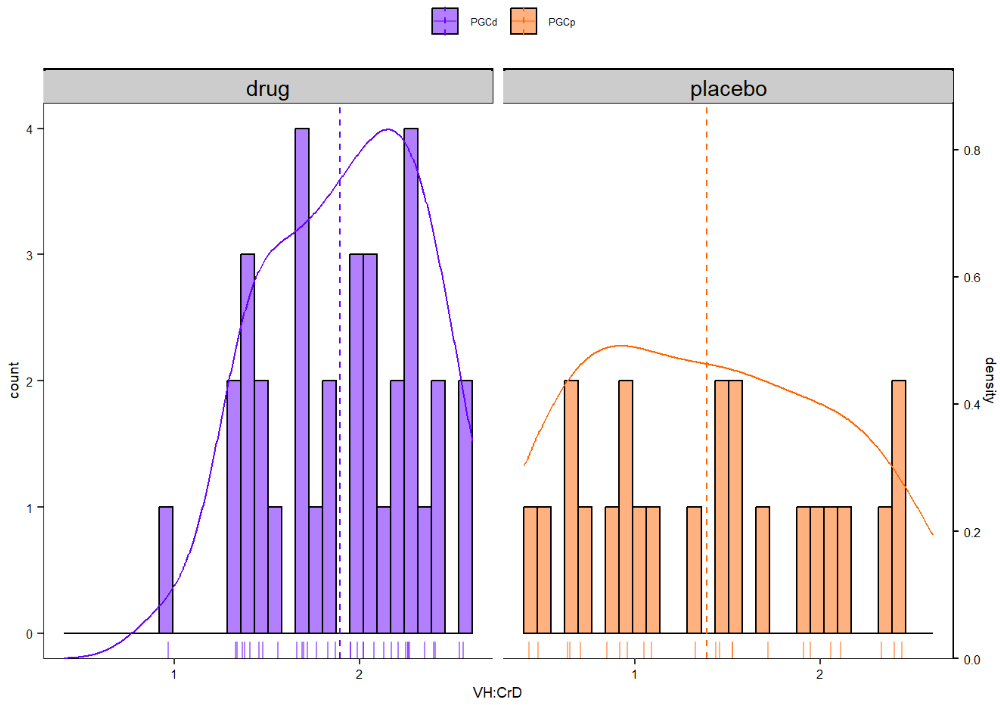

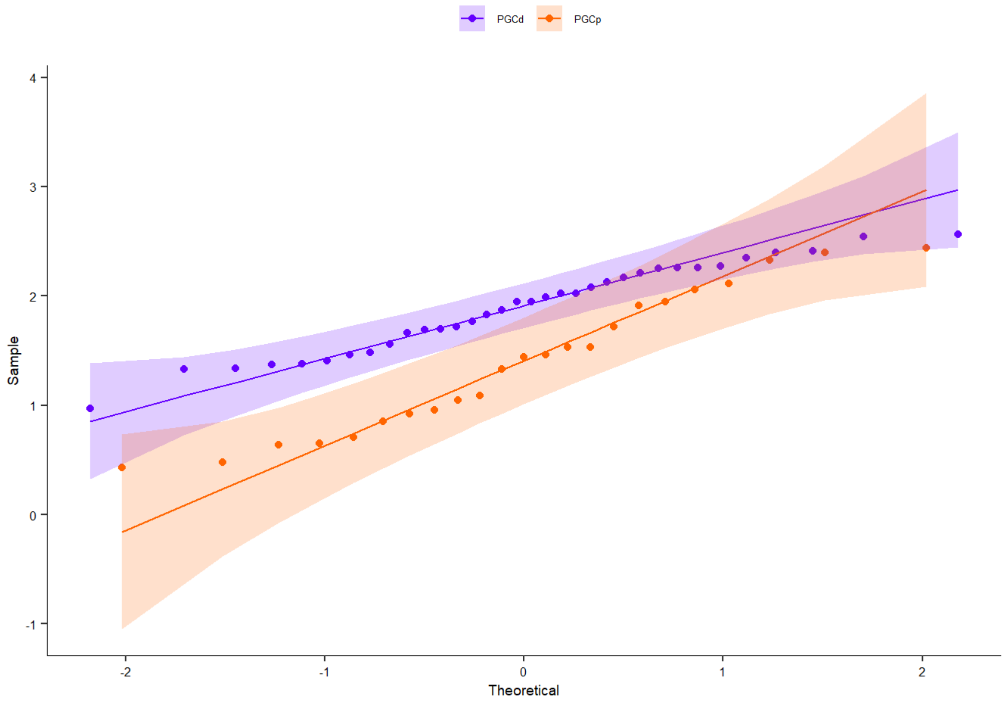

From the plot above, as all the points fall approximately along the reference line, we can assume normality.

3.2.2 Linear relationship between the dependent variable and covariate.

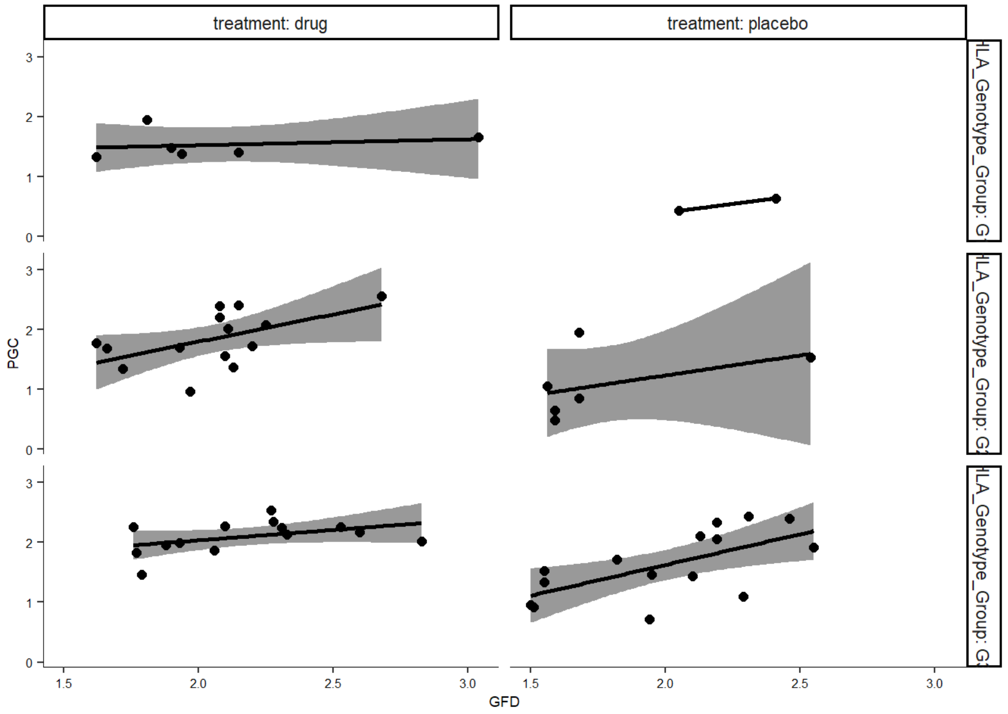

There was a linear relationship between the covariate (VH:CrD at GFD) and the outcome variable (VH:CrD at PGC) for each group, as assessed by visual inspection of a scatter plot.

3.2.3 Homogeneity of regression slopes.

```
## ANOVA Table (type II tests)
##
##                             Effect DFn DFd      F        p p<.05   ges
## 1                              GFD   1  45 15.125 3.29e-04     * 0.252
## 2                        treatment   1  45 18.160 1.02e-04     * 0.288
## 3               HLA_Genotype_Group   2  45 11.414 9.78e-05     * 0.337
## 4     treatment:HLA_Genotype_Group   2  45  1.998 1.48e-01       0.082
## 5                    GFD:treatment   1  45  1.122 2.95e-01       0.024
## 6           GFD:HLA_Genotype_Group   2  45  0.684 5.10e-01       0.029
## 7 GFD:treatment:HLA_Genotype_Group   2  45  0.751 4.78e-01       0.032
```

There was homogeneity of regression slopes as the interaction terms, between the covariate “GFD” (VH:CrD at GFD) and grouping variables (treatment and Genotype group), was not statistically significant, P = 0.478.

3.2.4 Normality of residuals.

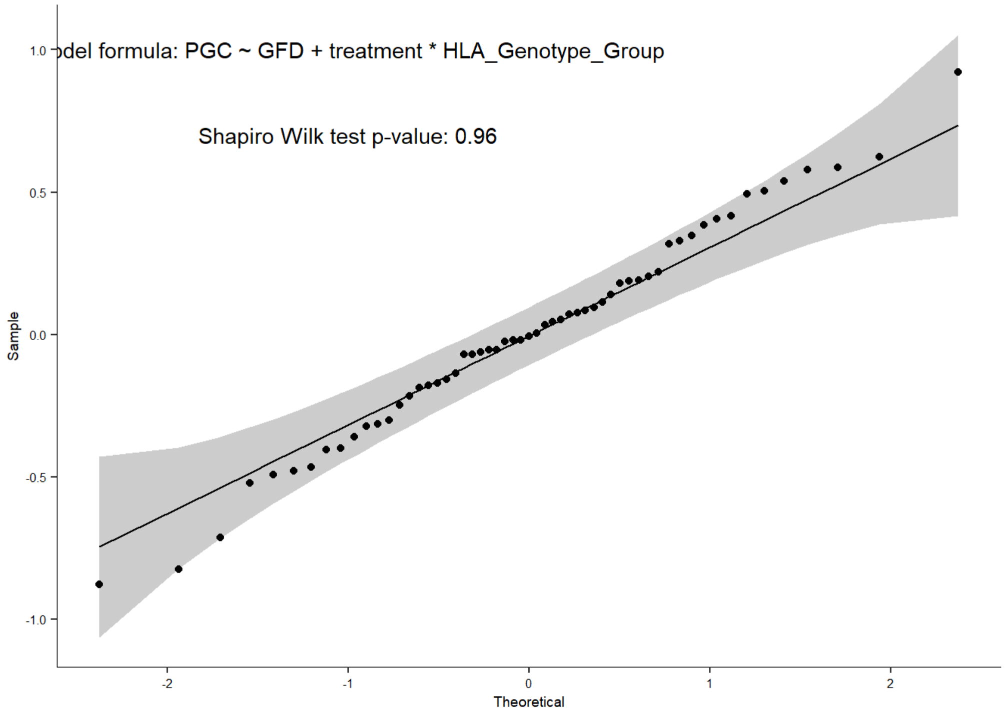

The Shapiro Wilk test was not significant P = 0.96, so we can assume normality of residuals

3.2.5 Homogeneity of variances

```
## # A tibble: 1 × 4
##     df1   df2 statistic     p
##   <int> <int>     <dbl> <dbl>
## 1     5    51      1.45 0.221
```

The Levene's test was not significant (P = 0.22), so we can assume homogeneity of the residual variances for all groups.

3.3 Computation of two-way ANCOVA

```
## ANOVA Table (type II tests)
##
##                       Effect DFn DFd      F        p p<.05   ges
## 1                        GFD   1  50 15.023 3.10e-04     * 0.231
## 2                  treatment   1  50 22.116 2.06e-05     * 0.307
## 3          HLA_Genotype_Group   2  50 10.849 1.22e-04     * 0.303
## 4 treatment:HLA_Genotype_Group   2  50  2.205 1.21e-01       0.081
```

After adjustment for the VH:CrD at GFD, there was no statistically significant interaction between treatment and the HLA-DQ genotype group on the histomorphometry parameters F(2,50) = 2.2, P = 0.121.

3.4 Post-hoc tests

3.4.1. Pairwise comparisons

```
## # A tibble: 3 × 10
##   HLA_Genotype_Group term    .y.   group1 group2    df statistic       p   p.adj
## * <fct>              <chr>   <chr> <chr>  <chr>  <dbl>     <dbl>   <dbl>   <dbl>
## 1 G1                 GFD*tr… PGC   drug   place…    50      3.40 0.00133 0.00133
## 2 G2                 GFD*tr… PGC   drug   place…    50      3.00 0.00425 0.00425
## 3 G3                 GFD*tr… PGC   drug   place…    50      2.45 0.0178  0.0178
## # ℹ 1 more variable: p.adj.signif <chr>
```

There was a statistically significant difference between the adjusted for GFD VH:CrD at PGC mean of drug and placebo group for all genotype groups.

3.4.2. Pairwise comparisons plot

Post-hoc pairwise multiple comparisons using estimated marginal means calculation (EMMs, also known as least-squares means) were conducted between the drug and placebo groups for the two-way ANCOVA. To address multiple testing, the Bonferroni correction was applied to P-values, total tests performed = 1. Statistical significance was defined as a P value adjusted < .05.

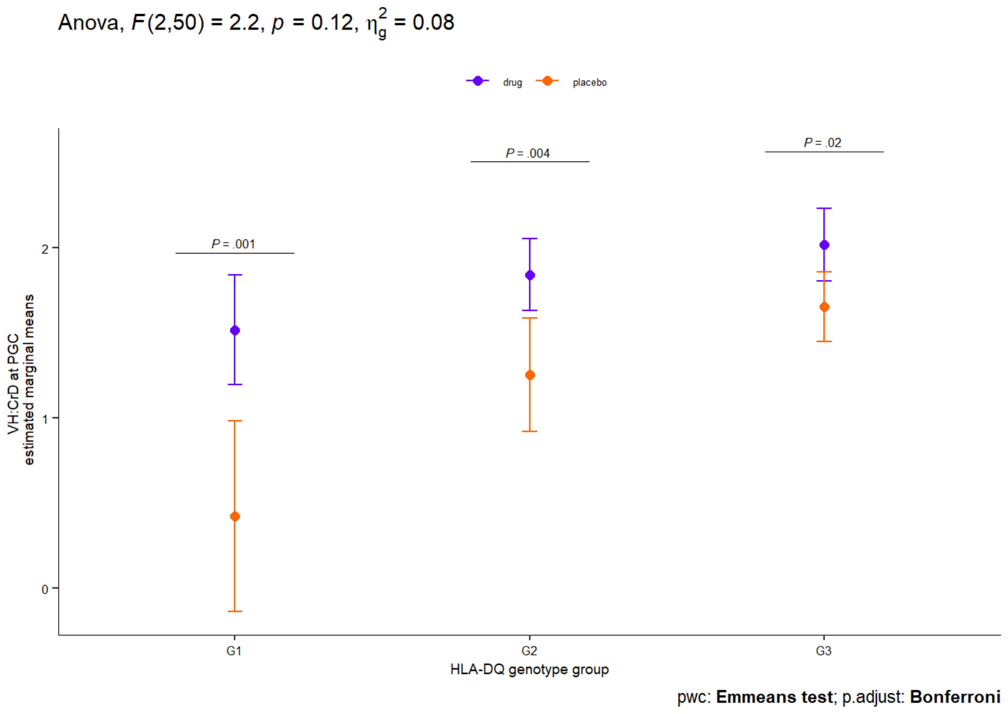

A two-way ANCOVA was performed with VH:CrD at PGC as a dependent variable and VH:CrD at GFD as covariate and Treatment (drug, placebo) and HLA-DQ genotype group (G1, G2, G3) as independent variables. ANCOVA, F (2,50) = 2.2, p = .12. Post-hoc pairwise multiple comparisons were performed between drug and placebo group among HLA-DQ genotype groups. VH:CrD ratio at PGC is shown as estimated marginal means±95% CI.

4. One-way ANCOVA (figure 4C)

For the one-way ANCOVA, only patients in the ***drug group*** (n = 34) were selected. The null hypothesis for this analysis was that there is no significant effect of HLA-DQ genetic background (represented by HLA-DQ genotype groups) on VH:CrD within the PGCd group, while adjusting for VH:CrD at GFDd. The one-way ANCOVA regression model included VH:CrD at PGCd as the dependent variable, VH:CrD at GFDd as a covariate, and HLA-DQ genotype group (G1, G2, G3) as independent variables.

4.1 Summary statistics

```
## # A tibble: 6 × 6
##   timepoint HLA_Genotype_Group variable     n  mean    sd
##   <fct>     <chr>              <fct>    <dbl> <dbl> <dbl>
## 1 GFD       G1                 VHCrD        6  2.08  0.5
## 2 PGC       G1                 VHCrD        6  1.53  0.23
## 3 GFD       G2                 VHCrD       14  2.05  0.27
## 4 PGC       G2                 VHCrD       14  1.84  0.46
## 5 GFD       G3                 VHCrD       14  2.17  0.33
## 6 PGC       G3                 VHCrD       14  2.1   0.27
```

4.2 Assumptions check

4.2.1 Normality assumption

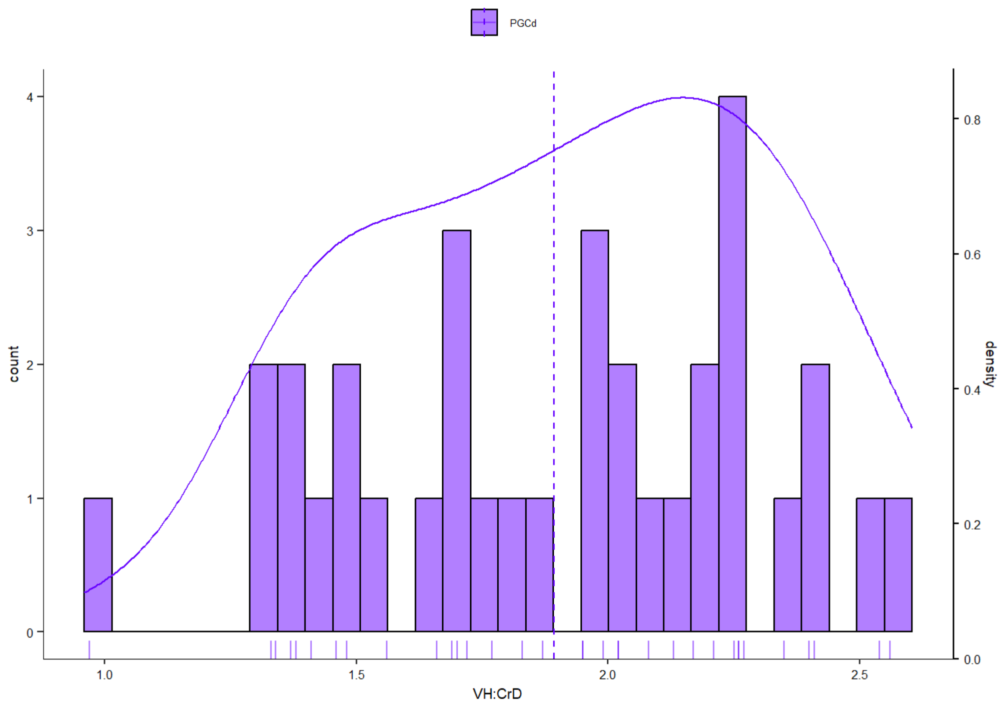

Histogram

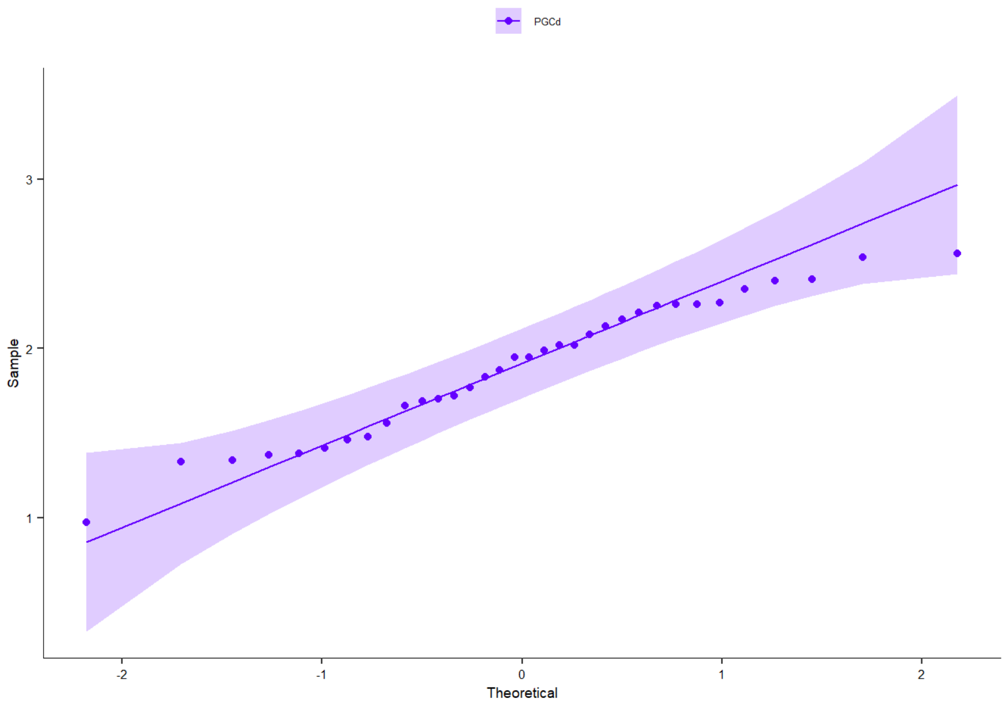

Q-Q plot

From the plot above, as all the points fall approximately along the reference line, we can assume normality.

4.2.2 Linear relationship between the dependent variable and covariate.

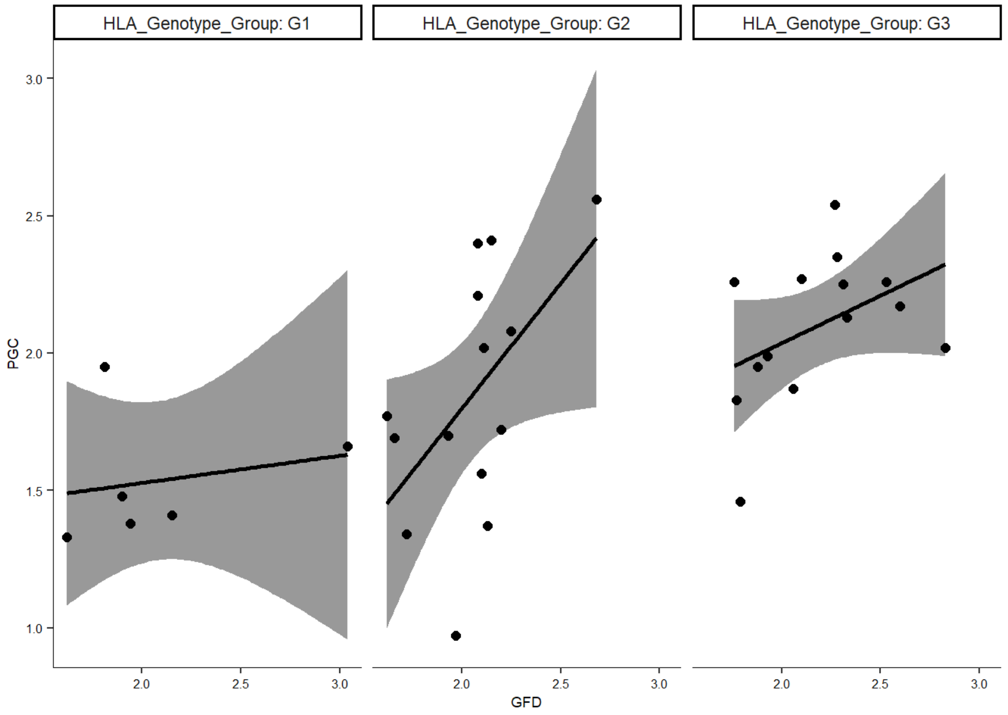

There was a linear relationship between the covariate (VH:CrD at GFD) and the outcome variable (VH:CrD at PGC) for each Genotype group, as assessed by visual inspection of a scatter plot.

4.2.3 Homogeneity of regression slopes.

```
## ANOVA Table (type II tests)
##
##                  Effect DFn DFd     F     p p<.05   ges
## 1                   GFD   1  28 5.717 0.024     * 0.170
## 2     HLA_Genotype_Group   2  28 5.360 0.011     * 0.277
## 3 GFD:HLA_Genotype_Group   2  28 1.728 0.196       0.110
```

There was homogeneity of regression slopes as the interaction terms, between the covariate "GFD" (VH:CrD at GFD) and grouping variable Genotype group, was not statistically significant, P = 0.196.

4.2.4 Normality of residuals.

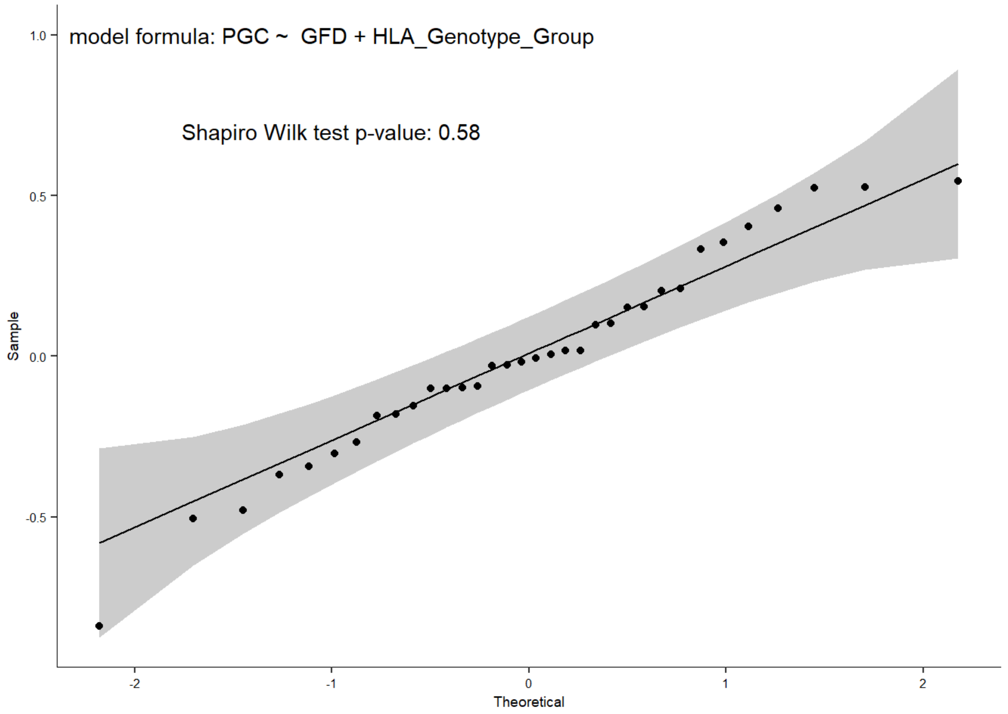

The Shapiro Wilk test was not significant P = 0.58, so we can assume normality of residuals

4.2.5 Homogeneity of variances

```
## # A tibble: 1 × 4
##     df1   df2 statistic     p
##   <int> <int>     <dbl> <dbl>
## 1     2    31      2.22 0.126
```

The Levene's test was not significant P = 0.13, so we can assume homogeneity of the residual variances for all groups.

4.3 Computation of one-way ANCOVA

```
## ANOVA Table (type II tests)
##
##                Effect DFn DFd     F     p p<.05   ges
## 1                 GFD   1  30 5.452 0.026     * 0.154
## 2  HLA_Genotype_Group   2  30 5.112 0.012     * 0.254
```

After adjustment for VH:CrD at GFD, there was a statistically significant difference in VH:CrD at PGC between the Genotype groups, F(2,30) = 5.1, P = 0.012.

4.4 Post-hoc tests

4.4.1. Pairwise comparisons

Pairwise comparisons were performed to identify which groups are different. Post-hoc pairwise multiple comparisons using estimated marginal means calculation (EMMs, also known as least-squares meansor adjusted means) between HLA-DQ genotype groups for the one-way ANCOVA. To address multiple testing, the Bonferroni correction was applied to P-values, total tests performed = 1 . Statistical significance was defined as a P value adjusted < .05.

```
## # A tibble: 3 × 9
##   term            .y.   group1 group2    df statistic       p  p.adj p.adj.signif
## * <chr>           <chr> <chr>  <chr>  <dbl>     <dbl>   <dbl>  <dbl> <chr>
## 1 GFD*HLA_Genot… PGC   G1     G2        30     -1.96 0.0598   0.179  ns
## 2 GFD*HLA_Genot… PGC   G1     G3        30     -3.18 0.00342 0.0102 *
## 3 GFD*HLA_Genot… PGC   G2     G3        30     -1.58 0.125    0.376  ns
```

4.4.2. Pairwise comparisons plot

```
## # A tibble: 3 × 8
##   group1 group2 estimate    se conf.low conf.high       p  p.adj
##   <chr>  <chr>     <dbl> <dbl>    <dbl>     <dbl>   <dbl>  <dbl>
## 1 G1     G2       -0.319 0.163   -0.653    0.0141 0.0598  0.179
## 2 G1     G3       -0.522 0.164   -0.857   -0.187  0.00342 0.0102
## 3 G2     G3       -0.202 0.128   -0.464    0.0597 0.125   0.376
```

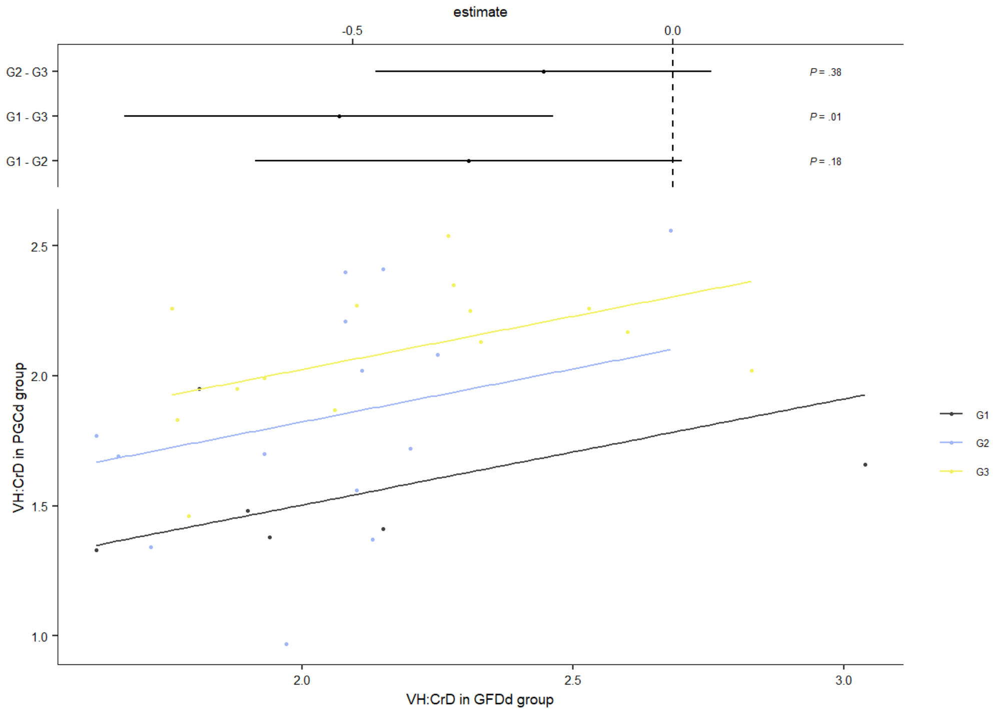

The estimated difference in the VH:CrD ratio for drug patients belonging to G3 genotypes versus G1 genotypes is -0.52 (95% CI -0.86 to -0.19), P.adj = 0.01.

Other estimated differences (G3-G2 and G2-G1) were not significant but showed the tendency of group G2 having the intermediate position between G1 and G3, when judging by the VH:CrD value.

5. Two-way ANCOVA (figure 4D)

To assess the interaction between treatment groups and HLA-DQ genetic backgrounds on Epithelial response to IFN-γ GSZ at PGC, a two-way ANCOVA was conducted using these values at PGC as the dependent variable, HLA-DQ genetic background (G1, G2, and G3 genotype groups) and treatment (placebo or drug) as independent variables, and baseline Epithelial response to IFN-γ GSZ (from GFD group) as a covariate. This analysis included 57 patients, with one subject from the placebo group excluded due to unidentified allele type. The study formulated two null hypotheses for the two-way ANCOVA analysis: 1) no Epithelial response to IFN-γ GSZ difference at PCG exists between treatment groups (placebo and drug), while accounting for Epithelial response to IFN-γ GSZ at GFD, and 2) no Epithelial response to IFN-γ GSZ differences at PCG exist across HLA-DQ genetic backgrounds (G1, G2, and G3 genotype groups), controlling for Epithelial response to IFN-γ GSZ at GFD.

5.1 Summary statistics

```
## # A tibble: 12 × 7
##    treatment timepoint HLA_Genotype_Group variable                 n  mean    sd
##    <fct>     <fct>     <chr>              <fct>                <dbl> <dbl> <dbl>
##  1 drug      GFD       G1                 epithelial_IFNg_res…     6 -0.27  1.42
##  2 drug      PGC       G1                 epithelial_IFNg_res…     6  1.08  2.85
##  3 drug      GFD       G2                 epithelial_IFNg_res…    14 -0.78  0.74
##  4 drug      PGC       G2                 epithelial_IFNg_res…    14 -0.39  1.11
##  5 drug      GFD       G3                 epithelial_IFNg_res…    14 -0.57  0.77
##  6 drug      PGC       G3                 epithelial_IFNg_res…    14 -0.44  1.15
##  7 placebo   GFD       G1                 epithelial_IFNg_res…     2  0.95  0.48
##  8 placebo   PGC       G1                 epithelial_IFNg_res…     2  3.92  0.28
##  9 placebo   GFD       G2                 epithelial_IFNg_res…     6 -1.4   1.39
## 10 placebo   PGC       G2                 epithelial_IFNg_res…     6  1.13  2.2
## 11 placebo   GFD       G3                 epithelial_IFNg_res…    15 -0.48  1.4
## 12 placebo   PGC       G3                 epithelial_IFNg_res…    14  1.62  2.17
```

5.2 Assumptions check

5.2.1 Normality assumption

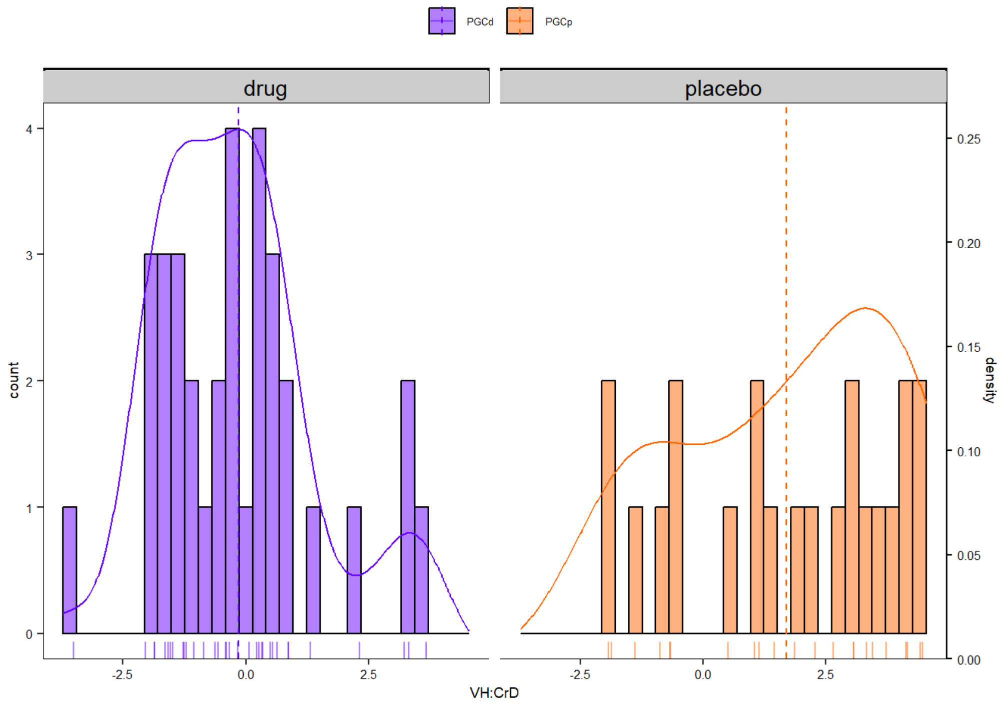

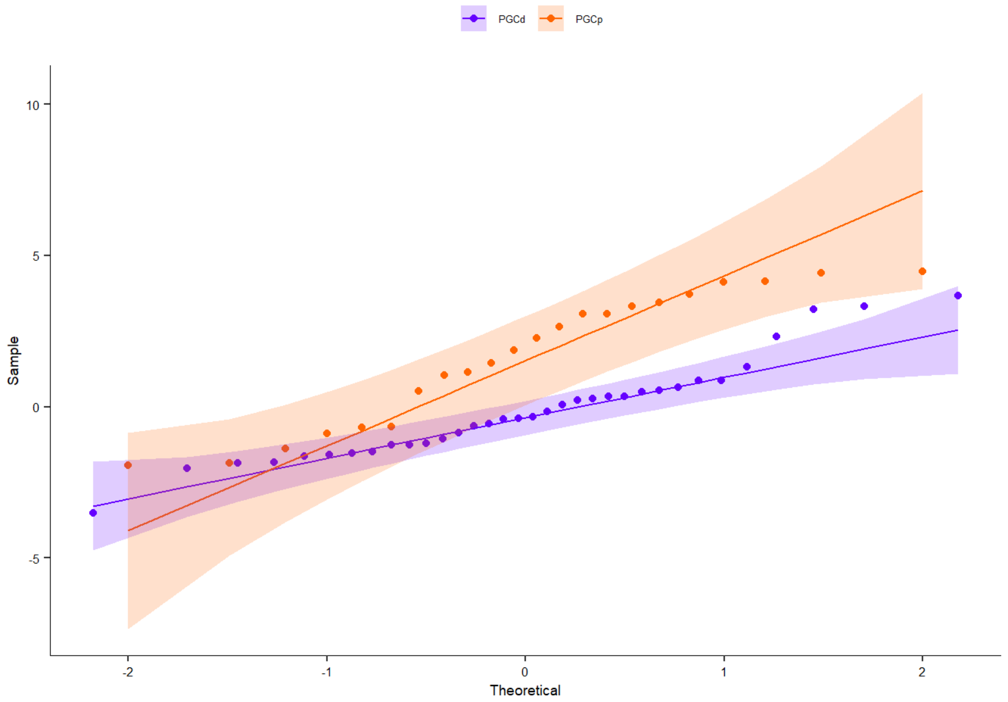

From the plot above, as all the points fall approximately along the reference line, we can assume normality.

5.2.2 Linear relationship between the dependent variable and covariate.

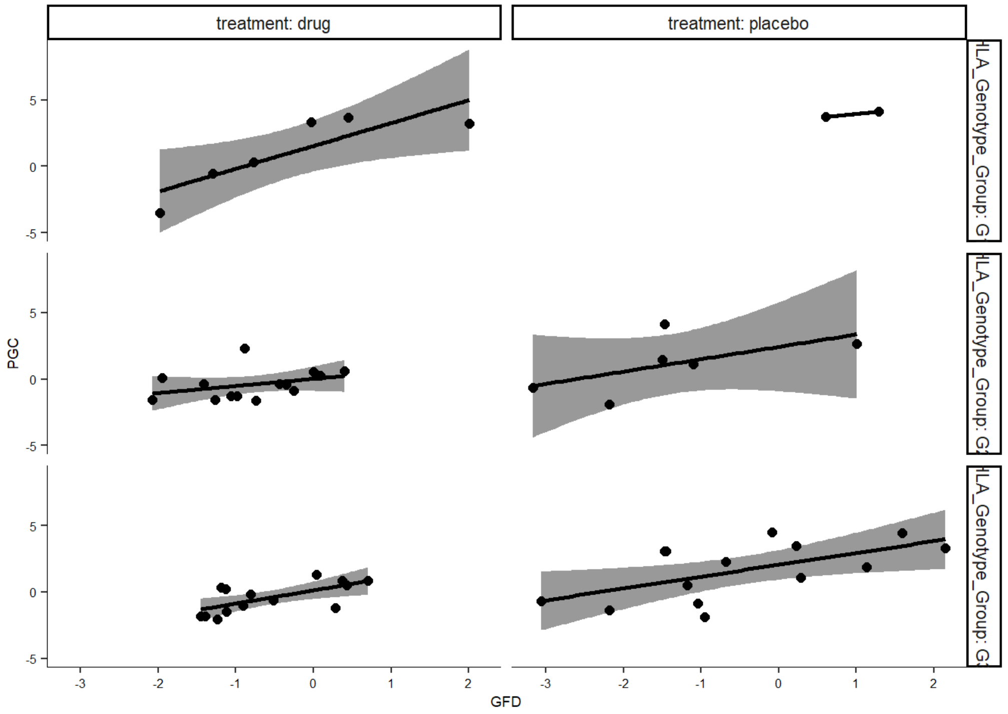

here was a linear relationship between the covariate (Epithelial response to IFN-γ GSZ at GFD) and the outcome variable (Epithelial response to IFN-γ GSZ at PGC) for each Genotype group, as assessed by visual inspection of a scatter plot.

5.2.3 Homogeneity of regression slopes.

```
## ANOVA Table (type II tests)
##
##                              Effect DFn DFd      F        p p<.05      ges
## 1                               GFD   1  44 30.516 1.68e-06     * 0.410000
## 2                         treatment   1  44 20.472 4.55e-05     * 0.318000
## 3                HLA_Genotype_Group   2  44  1.883 1.64e-01       0.079000
## 4      treatment:HLA_Genotype_Group   2  44  0.373 6.91e-01       0.017000
## 5                     GFD:treatment   1  44  0.028 8.67e-01       0.000646
## 6            GFD:HLA_Genotype_Group   2  44  1.286 2.87e-01       0.055000
## 7 GFD:treatment:HLA_Genotype_Group   2  44  0.232 7.94e-01       0.010000
```

There was homogeneity of regression slopes as the interaction terms, between the covariate “GFD” (Epithelial response to IFN-γ GSZ at GFD) and grouping variables (treatment and Genotype group), was not statistically significant.

5.2.4 Normality of residuals.

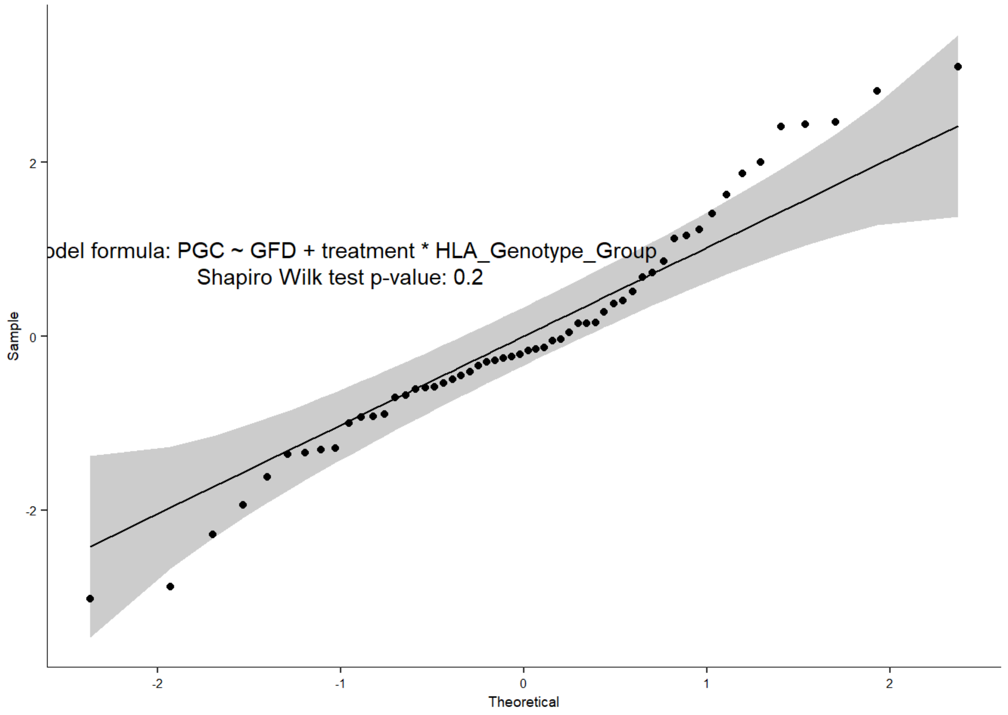

The Shapiro Wilk test was not significant P = 0.2, so we can assume normality of residuals

5.2.5 Homogeneity of variances

```
## # A tibble: 1 × 4
##     df1   df2 statistic      p
##   <int> <int>     <dbl>  <dbl>
## 1     5    50      2.05 0.0876
```

The Levene's test was not significant (P = 0.09), so we can assume homogeneity of the residual variances for all groups.

5.3 Computation of two-way ANCOVA

```
## ANOVA Table (type II tests)
##
##                         Effect DFn DFd      F        p p<.05   ges
## 1                          GFD   1  49 31.475 9.27e-07     * 0.391
## 2                    treatment   1  49 25.344 6.88e-06     * 0.341
## 3           HLA_Genotype_Group   2  49  1.974 1.50e-01       0.075
## 4 treatment:HLA_Genotype_Group   2  49  0.071 9.31e-01       0.003
```

After adjustment for the Epithelial response to IFN-γ GSZ at GFD, there was no statistically significant interaction between treatment and the HLA-DQ genotype group on the IFN-γ responce F(2,49) = 0.1, P = 0.931.

5.4 Post-hoc tests

5.4.2. Pairwise comparisons

```
## # A tibble: 3 × 10
##   HLA_Genotype_Group term    .y.   group1 group2    df statistic       p   p.adj
## * <fct>              <chr>   <chr> <chr>  <chr>  <dbl>     <dbl>   <dbl>   <dbl>
## 1 G1                 GFD*tr… PGC   drug   place…    49     -1.39 1.71e-1 1.71e-1
## 2 G2                 GFD*tr… PGC   drug   place…    49     -3.08 3.39e-3 3.39e-3
## 3 G3                 GFD*tr… PGC   drug   place…    49     -3.71 5.31e-4 5.31e-4
## # ℹ 1 more variable: p.adj.signif <chr>
```

There was a statistically significant difference between the adjusted for GFD Epithelial response to IFN-γ GSZ at PGC mean of drug and placebo group for G2 (P = 0.003) and G3 genotype groups (P = 5e-04).

5.4.3. Pairwise comparisons plot

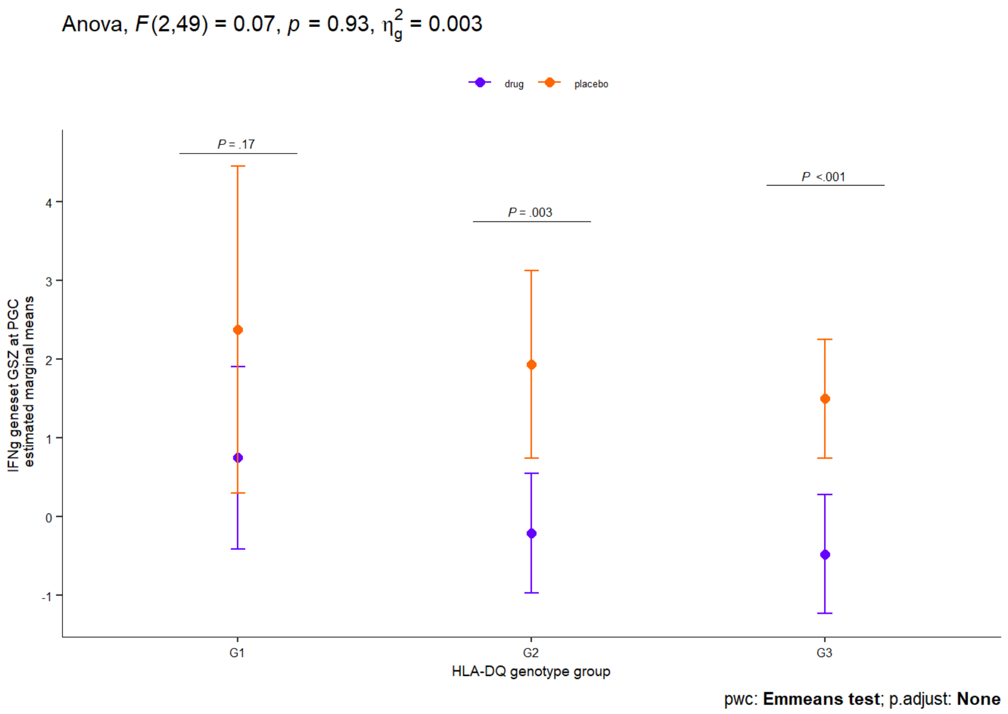

A two-way ANCOVA plot, examining the effects of treatment and HLA-DQ genetic background on post-gluten challenge epithelial-IFN-γ-GSZ-Score. Anova, F (2,49) = 0.07, p = .93.

6. One-way ANCOVA (figure S4A)

For the one-way ANCOVA, only patients in the *** drug group *** (n = 34) were selected. The null hypothesis for this analysis was that there is no significant effect of HLA-DQ genetic background (represented by HLA-DQ genotype groups) on VH within the PGCd group, while adjusting for VH at GFDd. The one-way ANCOVA regression model included VH at PGCd as the dependent variable, VH at GFDd as a

covariate, and HLA-DQ genotype group (G1, G2, G3) as independent variables.

6.1 Summary statistics

```
## # A tibble: 6 × 6
##   timepoint HLA_Genotype_Group variable     n  mean    sd
##   <fct>     <chr>              <fct>    <dbl> <dbl> <dbl>
## 1 GFD       G1                 VH           6  343.  36.0
## 2 PGC       G1                 VH           6  292.  29.2
## 3 GFD       G2                 VH          14  369.  39.5
## 4 PGC       G2                 VH          14  362.  47.1
## 5 GFD       G3                 VH          14  383.  44.4
## 6 PGC       G3                 VH          14  370.  34.4
```

6.2 Assumptions check

6.2.1 Normality assumption

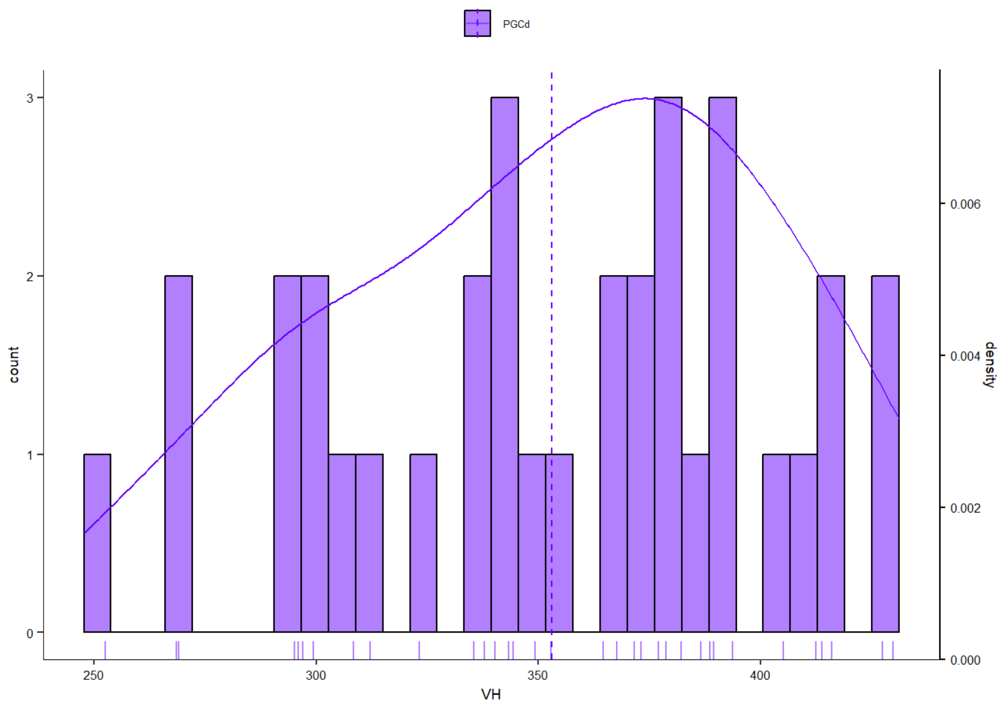

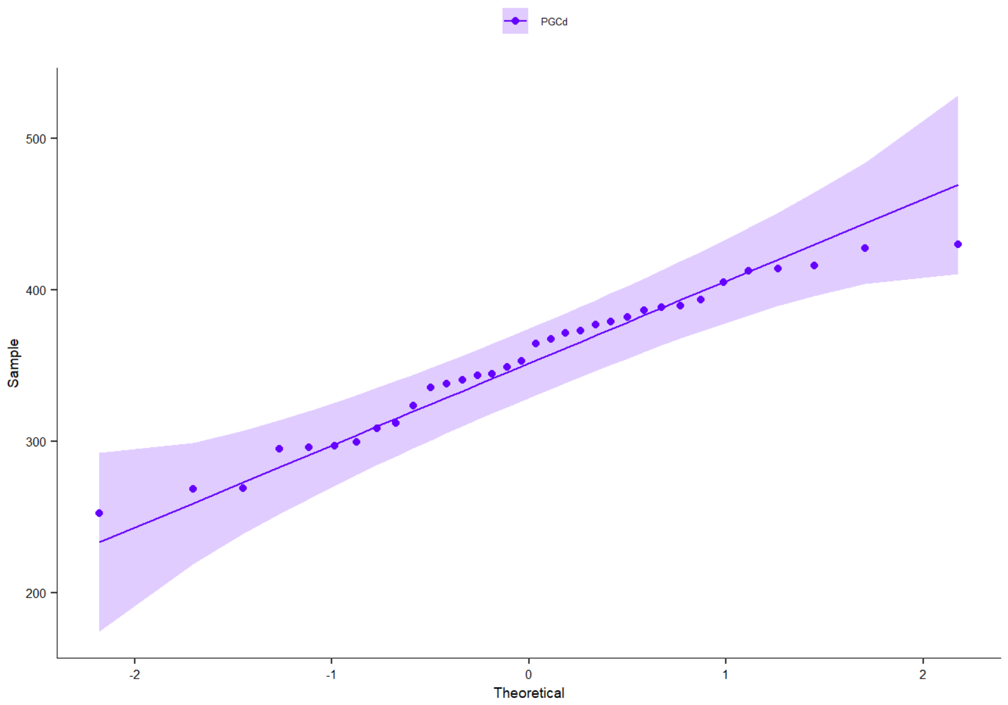

From the plot above, as all the points fall approximately along the reference line, we can assume normality.

6.2.2 Linear relationship between the dependent variable and covariate.

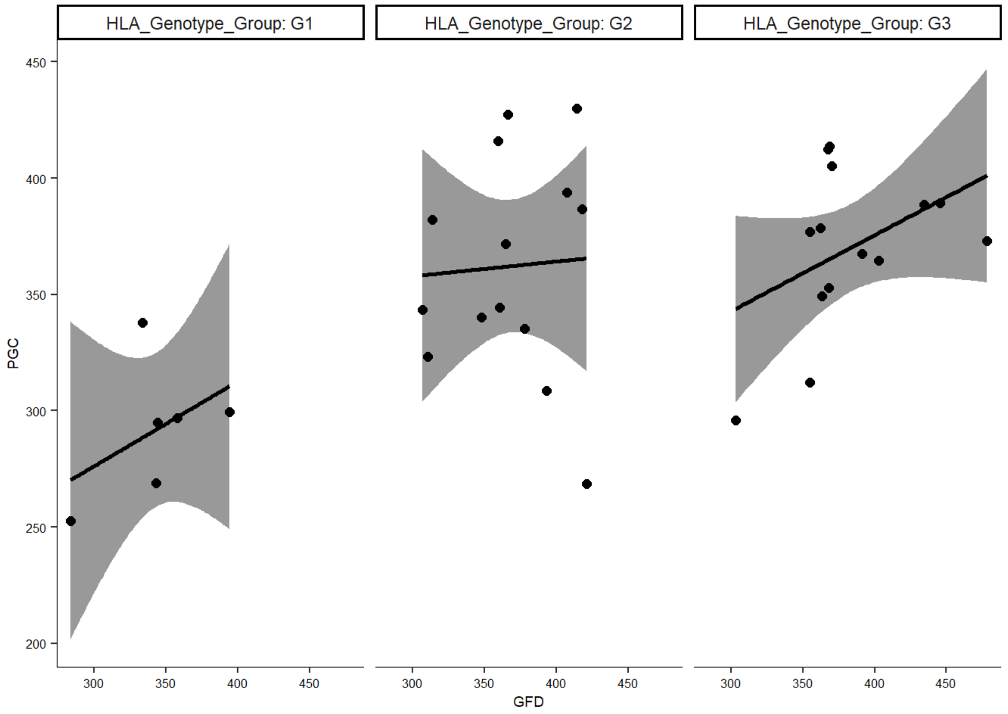

There was a linear relationship between the covariate (VH at GFD) and the outcome variable (VH at PGC) for each Genotype group, as assessed by visual inspection of a scatter plot.

6.2.3 Homogeneity of regression slopes.

```
## ANOVA Table (type II tests)
##
##                  Effect DFn DFd     F     p p<.05   ges
## 1                   GFD   1  28 1.734 0.199       0.058
## 2    HLA_Genotype_Group   2  28 6.245 0.006     * 0.308
## 3 GFD:HLA_Genotype_Group   2  28 0.289 0.751       0.020
```

There was homogeneity of regression slopes as the interaction terms, between the covariate “GFD” (VH at GFD) and grouping variable Genotype group, was not statistically significant, P = 0.751.

6.2.4 Normality of residuals.

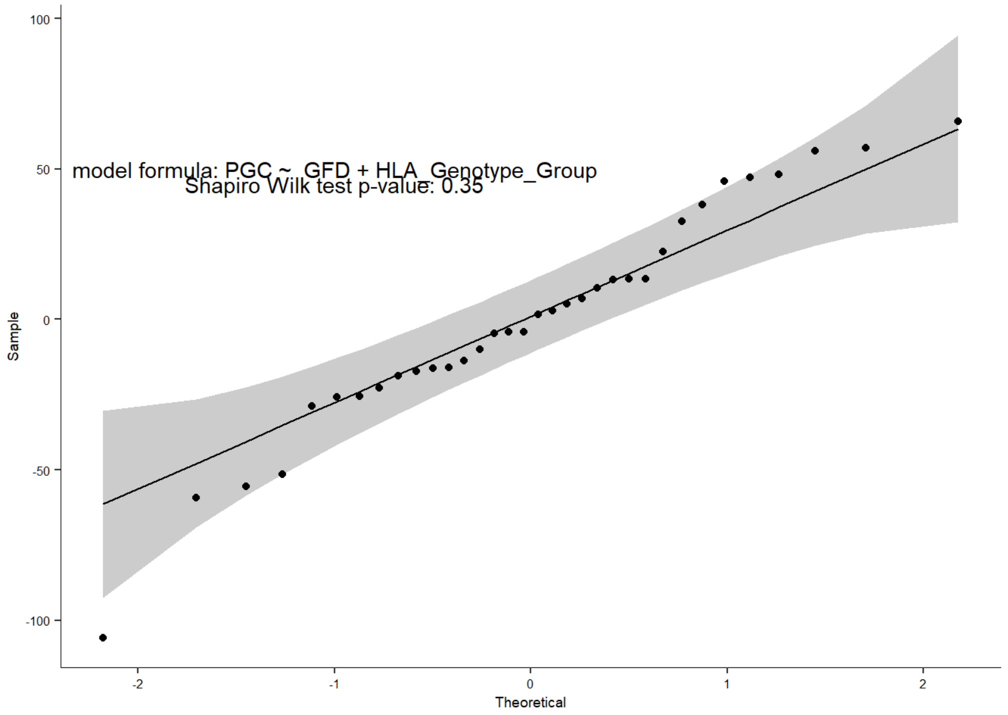

The Shapiro Wilk test was not significant (P = 0.35), so we can assume normality of residuals

6.2.5 Homogeneity of variances

```
## # A tibble: 1 × 4
##     df1   df2 statistic     p
##   <int> <int>     <dbl> <dbl>
## 1     2    31      1.77 0.187
```

The Levene's test was not significant (P = 0.19), so we can assume homogeneity of the residual variances for all groups.

6.3 Computation of one-way ANCOVA

```
## ANOVA Table (type II tests)
##
##              Effect DFn DFd     F     p p<.05   ges
## 1               GFD   1  30 1.820 0.187       0.057
## 2 HLA_Genotype_Group   2  30 6.556 0.004     * 0.304
```

After adjustment for the VH at GFD, there was statistically significant difference in VH at PGC score between the HLA-DQ genotype groups, F(2,30) = 6.6, P = 0.004.

6.4 Post-hoc tests

6.4.1. Pairwise comparisons

```
## # A tibble: 3 × 9
##   term          .y.   group1 group2    df statistic       p    p.adj p.adj.signif
## * <chr>         <chr> <chr>  <chr>  <dbl>     <dbl>   <dbl>    <dbl> <chr>
## 1 GFD*HLA_Geno… PGC   G1     G2        30     -3.30  0.00251 0.00752 **
## 2 GFD*HLA_Geno… PGC   G1     G3        30     -3.41  0.00189 0.00566 **
## 3 GFD*HLA_Geno… PGC   G2     G3        30     -0.300 0.766   1       ns
```

6.4.2. Pairwise comparisons plot

```
## # A tibble: 3 × 9
##   term            .y.   group1 group2 estimate conf.low conf.high       p   p.adj
##   <chr>           <chr> <chr>  <chr>     <dbl>    <dbl>     <dbl>   <dbl>   <dbl>
## 1 GFD*HLA_Genot… PGC   G1     G2        -64.5    -104.      -24.6 0.00251 0.00752
## 2 GFD*HLA_Genot… PGC   G1     G3        -69.0    -110.      -27.6 0.00189 0.00566
## 3 GFD*HLA_Genot… PGC   G2     G3         -4.50    -35.1      26.1 0.766   1
```

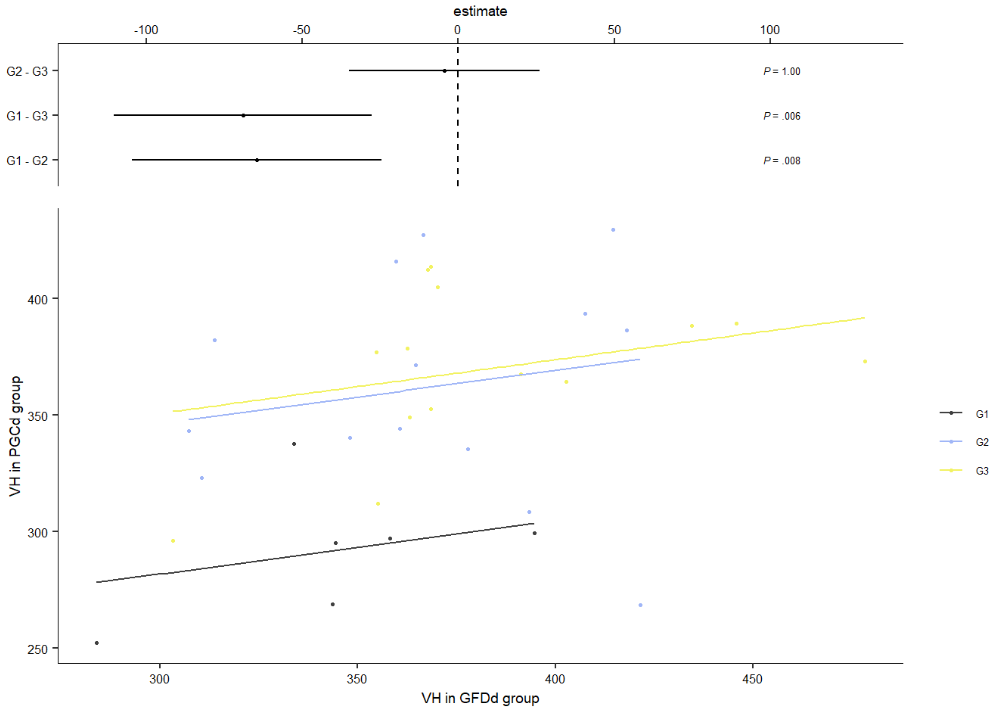

The estimated difference in the VH for drug patients belonging to G3 genotypes versus G1 genotypes is -69(95% CI -110.35 to -27.65), P.adj = 0.01.

The estimated difference in the VH for drug patients belonging to G2 genotypes versus G1 genotypes is -64.5(95% CI -104.44 to -24.57), P.adj = 0.01.

Other estimated difference (G3-G2) was not significant.

7. One-way ANCOVA (figure S4B)

For the one-way ANCOVA, only patients in the drug group (n = 34) were selected. The null hypothesis for this analysis was that there is no significant effect of HLA-DQ genetic background (represented by HLA-DQ genotype groups) on CrD within the PGCd group, while adjusting for CrD at GFDd. The one-way ANCOVA regression model included CrD at PGCd as the dependent variable, CrD at GFDd as a covariate, and HLA-DQ genotype group (G1, G2, G3) as independent variables.

7.1 Summary statistics

```
## # A tibble: 6 × 6
##   timepoint HLA_Genotype_Group variable     n  mean    sd
##   <fct>     <chr>              <fct>    <dbl> <dbl> <dbl>
## 1 GFD       G1                 CrD          6  171.  33.5
## 2 PGC       G1                 CrD          6  192.  20.2
## 3 GFD       G2                 CrD         14  181.  18.1
## 4 PGC       G2                 CrD         14  204.  35.6
## 5 GFD       G3                 CrD         14  178.  16.8
## 6 PGC       G3                 CrD         14  178.  19.3
```

7.2 Assumptions check

7.2.1 Normality assumption

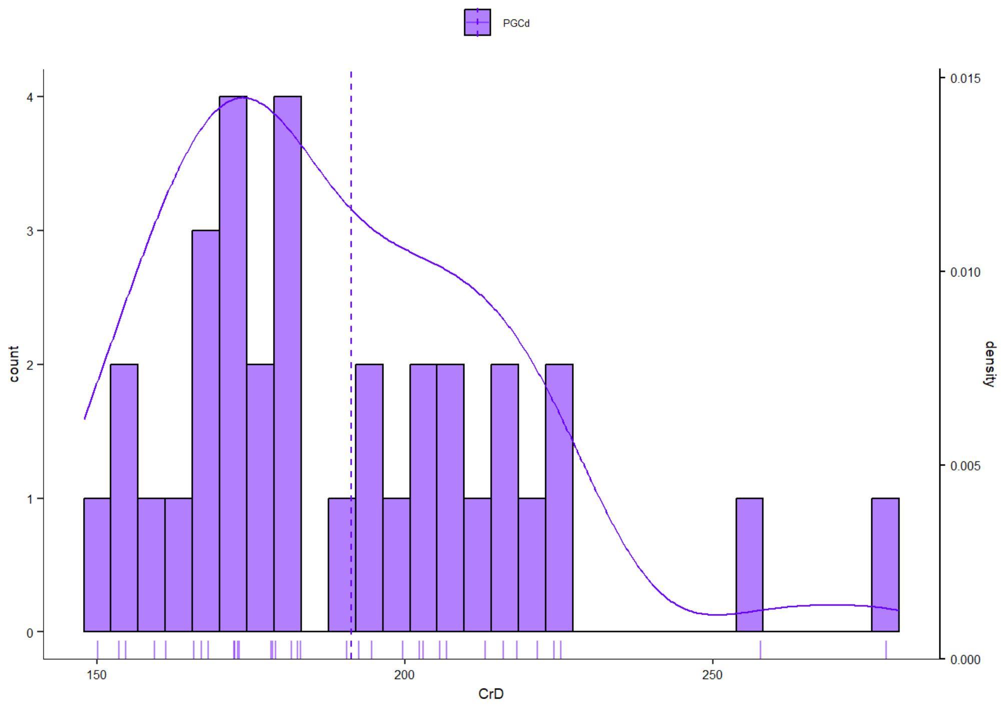

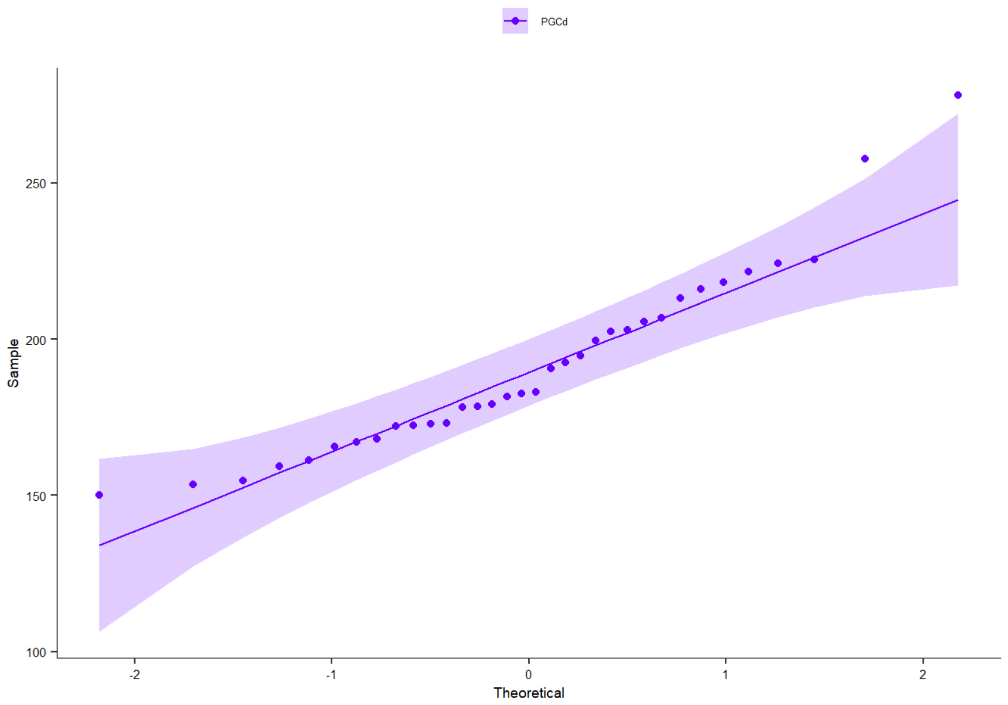

From the plot above, as all the points fall approximately along the reference line, we can assume normality.

7.2.2 Linear relationship between the dependent variable and covariate.

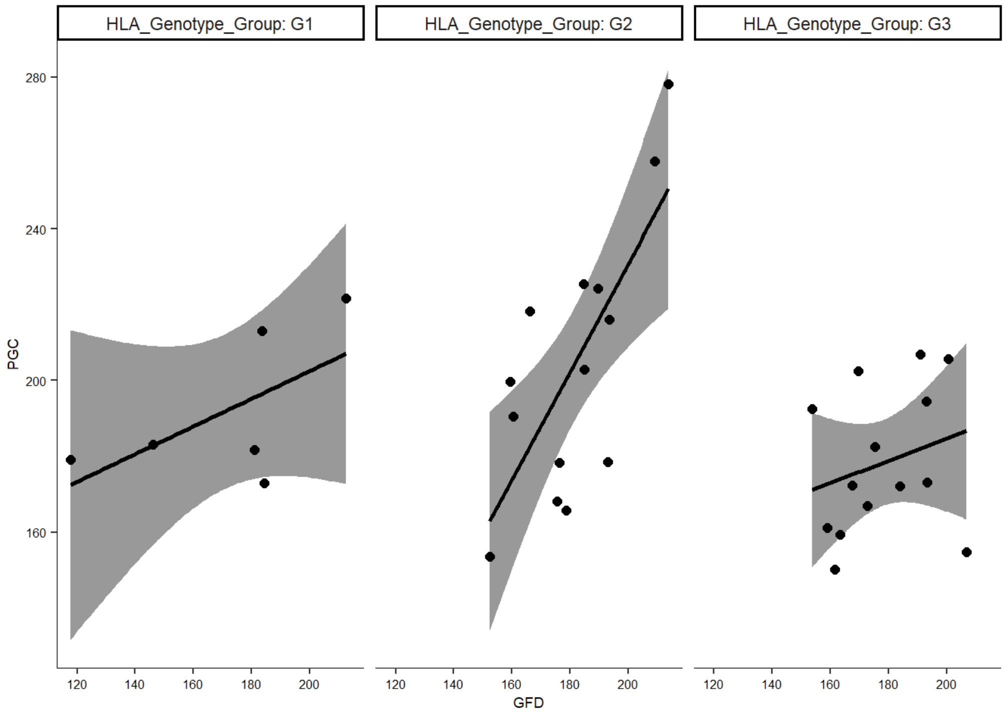

There was a linear relationship between the covariate (CrD at GFD) and the outcome variable (CrD at PGC) for each Genotype group, as assessed by visual inspection of a scatter plot.

7.2.3 Homogeneity of regression slopes.

```
## ANOVA Table (type II tests)
##
##                   Effect DFn DFd      F     p p<.05   ges
## 1                    GFD   1  28 12.715 0.001     * 0.312
## 2     HLA_Genotype_Group   2  28  4.205 0.025     * 0.231
## 3 GFD:HLA_Genotype_Group   2  28  3.534 0.043     * 0.202
```

Homogeneity of regression slopes assumption is violated as the interaction terms, between the covariate "GFD" (CrD at GFD) and grouping variable Genotype group, was statistically significant, p = 0.043.

7.2.4 Normality of residuals.

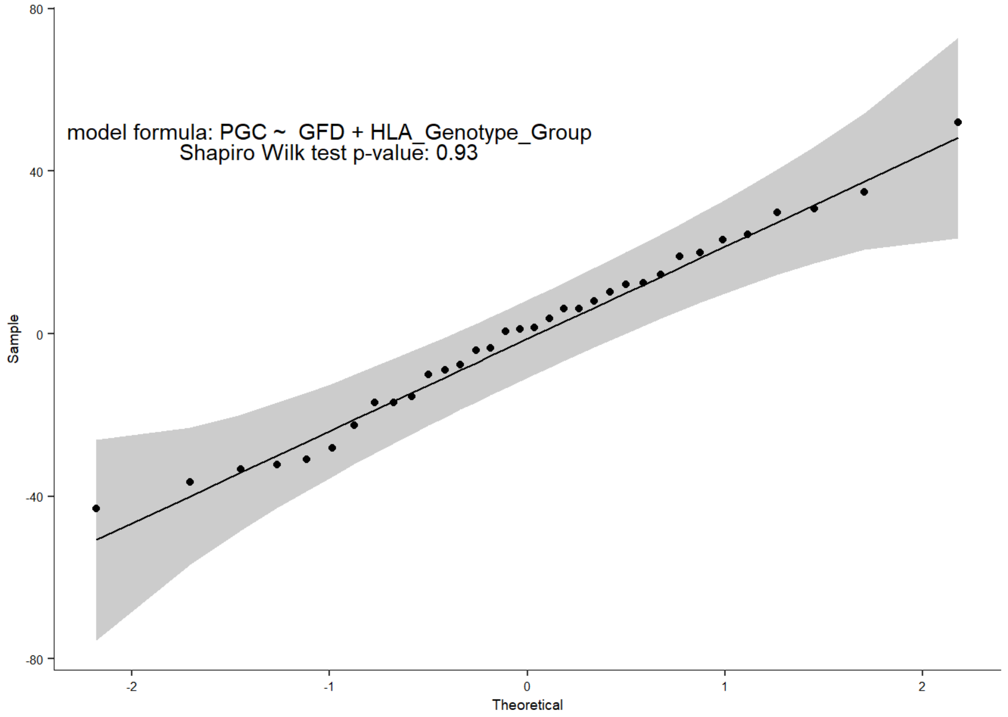

The Shapiro Wilk test was not significant (p = 0.93), so we can assume normality of residuals

7.2.5 Homogeneity of variances

```
## # A tibble: 1 × 4
##     df1   df2 statistic     p
##   <int> <int>     <dbl> <dbl>
## 1     2    31      1.35 0.273
```

The Levene's test was not significant (p = 0.27), so we can assume homogeneity of the residual variances for all groups.

7.3 Computation of one-way ANCOVA

```
## ANOVA Table (type II tests)
##
##               Effect DFn DFd      F     p p<.05   ges
## 1                GFD   1  30 10.877 0.003     * 0.266
## 2 HLA_Genotype_Group   2  30  3.597 0.040     * 0.193
```

After adjustment for CrD at GFD, there was a statistically significant difference in CrD at PGC between the Genotype groups, F(2, 30) = 3.597, p = 0.04.

7.4 Post-hoc tests

7.4.1. Pairwise comparisons

```
## # A tibble: 3 × 9
##   term             .y.   group1 group2    df statistic      p  p.adj p.adj.signif
## * <chr>            <chr> <chr>  <chr>  <dbl>     <dbl>  <dbl>  <dbl> <chr>
## 1 GFD*HLA_Genoty… PGC   G1     G2        30    -0.437 0.666  1      ns
## 2 GFD*HLA_Genoty… PGC   G1     G3        30     1.57  0.127  0.382  ns
## 3 GFD*HLA_Genoty… PGC   G2     G3        30     2.60  0.0142 0.0425 *
```

7.4.2. Pairwise comparisons plot

```
## # A tibble: 3 × 9
##   term              .y.   group1 group2 estimate conf.low conf.high      p  p.adj
##   <chr>             <chr> <chr>  <chr>     <dbl>    <dbl>     <dbl>  <dbl>  <dbl>
## 1 GFD*HLA_Genotyp… PGC   G1     G2        -5.17   -29.4       19.0 0.666  1
## 2 GFD*HLA_Genotyp… PGC   G1     G3        18.4     -5.57      42.4 0.127  0.382
## 3 GFD*HLA_Genotyp… PGC   G2     G3        23.6      5.09      42.1 0.0142 0.0425
```

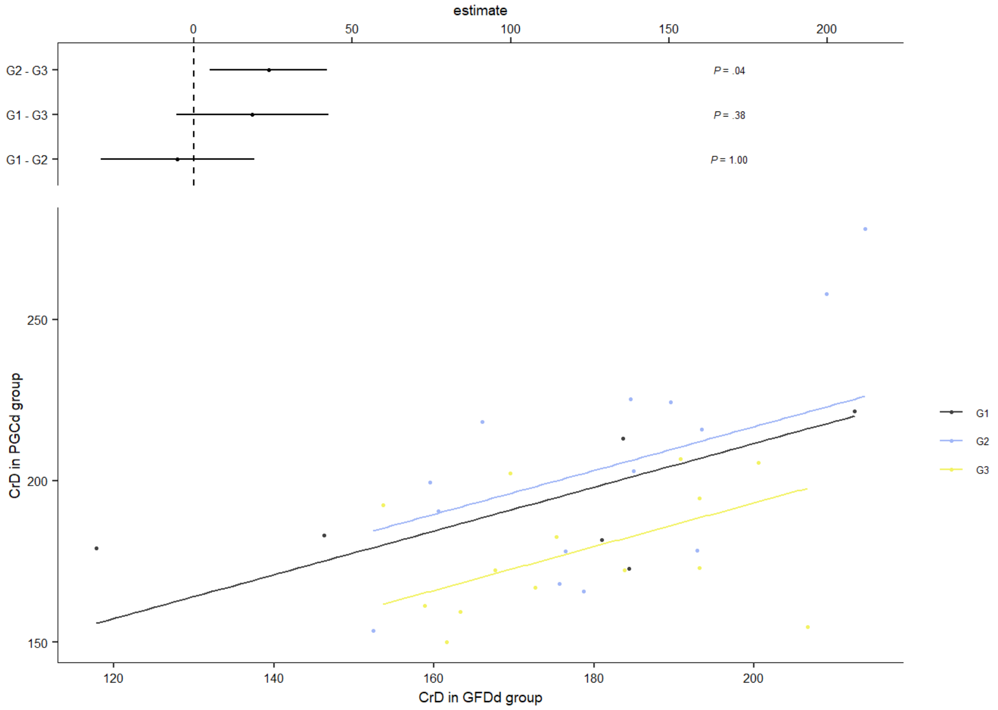

The estimated difference in the CrD for drug patients belonging to G2 genotypes versus G3 genotypes is 23.6 (95% CI 5.09 to 42.08), P.adj = 0.04.

Other estimated differences (G1-G2 and G1-G3) were not significant.

8. Session information.

```
## R version 4.3.0 (2023-04-21 ucrt)
## Platform: x86_64-w64-mingw32/x64 (64-bit)
## Running under: Windows 10 x64 (build 19045)
## 
## Matrix products: default
## 
## 
## locale:
## [1] LC_COLLATE=Ukrainian_Ukraine.utf8  LC_CTYPE=Ukrainian_Ukraine.utf8   
## [3] LC_MONETARY=Ukrainian_Ukraine.utf8 LC_NUMERIC=C                      
## [5] LC_TIME=Ukrainian_Ukraine.utf8    
## 
## time zone: Europe/Kiev
## tzcode source: internal
## 
## attached base packages:
## [1] stats4    stats     graphics  grDevices utils     datasets  methods  
## [8] base     
## 
## other attached packages:
##  [1] DT_0.29                     gridExtra_2.3              
##  [3] lazyWeave_3.0.2             readxl_1.4.3               
##  [5] ggthemes_4.2.4              ggplotify_0.1.2            
##  [7] numform_0.7.0               cowplot_1.1.1              
##  [9] data.table_1.14.8           ggpubr_0.6.0               
## [11] emmeans_1.8.8               DESeq2_1.41.2              
## [13] SummarizedExperiment_1.31.1 Biobase_2.61.0             
## [15] MatrixGenerics_1.13.1       matrixStats_1.0.0          
## [17] GenomicRanges_1.53.1        GenomeInfoDb_1.37.4        
## [19] IRanges_2.35.1              S4Vectors_0.39.1           
## [21] BiocGenerics_0.47.0         rstatix_0.7.2              
## [23] dplyr_1.1.2                 reshape2_1.4.4             
## [25] ggplot2_3.4.3              
## 
## loaded via a namespace (and not attached):
##  [1] bitops_1.0-7            sandwich_3.0-2          rlang_1.1.1            
##  [4] magrittr_2.0.3          multcomp_1.4-25         compiler_4.3.0         
##  [7] mgcv_1.8-42             vctrs_0.6.3             stringr_1.5.0          
## [10] pkgconfig_2.0.3         crayon_1.5.2            fastmap_1.1.1          
## [13] ellipsis_0.3.2          backports_1.4.1         XVector_0.41.1         
## [16] labeling_0.4.3          utf8_1.2.3              rmarkdown_2.25         
## [19] purrr_1.0.2             xfun_0.40               zlibbioc_1.47.0        
## [22] cachem_1.0.8            jsonlite_1.8.7          DelayedArray_0.27.5    
## [25] BiocParallel_1.35.2     broom_1.0.5             parallel_4.3.0         
## [28] R6_2.5.1                bslib_0.5.1             stringi_1.7.12         
## [31] car_3.1-2               jquerylib_0.1.4         cellranger_1.1.0       
## [34] estimability_1.4.1      Rcpp_1.0.11             knitr_1.44             
## [37] zoo_1.8-12              Matrix_1.5-4.1          splines_4.3.0          
## [40] tidyselect_1.2.0        rstudioapi_0.15.0       abind_1.4-5            
## [43] yaml_2.3.7              codetools_0.2-19        lattice_0.21-8         
## [46] tibble_3.2.1            plyr_1.8.8              withr_2.5.0            
## [49] coda_0.19-4             evaluate_0.21           gridGraphics_0.5-1     
## [52] survival_3.5-5          pillar_1.9.0            carData_3.0-5          
## [55] generics_0.1.3          RCurl_1.98-1.12         munsell_0.5.0          
## [58] scales_1.2.1            xtable_1.8-4            glue_1.6.2             
## [61] tools_4.3.0             locfit_1.5-9.8          ggsignif_0.6.4         
## [64] mvtnorm_1.2-3           grid_4.3.0              tidyr_1.3.0            
## [67] crosstalk_1.2.0         colorspace_2.1-0        nlme_3.1-162           
## [70] GenomeInfoDbData_1.2.10 cli_3.6.1               fansi_1.0.4            
## [73] S4Arrays_1.1.4          gtable_0.3.4            yulab.utils_0.0.9      
## [76] sass_0.4.7              digest_0.6.31           SparseArray_1.1.10     
## [79] TH.data_1.1-2           farver_2.1.1            htmlwidgets_1.6.2      
## [82] memoise_2.0.1           htmltools_0.5.6         lifecycle_1.0.3        
## [85] MASS_7.3-60
```
